# Galectin-3 exacerbates autoimmune diabetes by limiting regulatory T cell differentiation and function

**DOI:** 10.1126/sciadv.adz7916

**Published:** 2026-01-01

**Authors:** Lingxiang Xie, Rong Zhang, Hailan Zou, Jingyi Hu, Jiangming Deng, Jin Ding, Long Liu, Qiuqiu Lin, Bin Zhao, Aimin Xu, Zhiguang Zhou, Yang Xiao

**Affiliations:** ^1^ National Clinical Research Center for Metabolic Diseases, Key Laboratory of Diabetes Immunology, Ministry of Education, and Department of Metabolism and Endocrinology, The Second Xiangya Hospital of Central South University, Changsha, China.; ^2^ Department of Metabolism and Endocrinology, The Fourth of Hospital of Changsha, Changsha, Hunan, China.; ^3^ State Key Laboratory of Pharmaceutical Biotechnology, The University of Hong Kong, Hong Kong, China.; ^4^ Department of Medicine, The University of Hong Kong, Hong Kong, China.; ^5^ Department of Pharmacology and Pharmacy, The University of Hong Kong, Hong Kong, China.

## Abstract

Galectin-3, a β-galactoside–binding lectin, has been implicated in several inflammatory and autoimmune diseases. However, the significance of circulating Galectin-3 in type 1 diabetes (T1D) remains unclear. Here, we report that compared to healthy controls, patients with T1D and their first-degree relatives (FDRs) exhibited significantly increased serum Galectin-3 levels primarily produced and secreted by monocytes/macrophages. Pharmacological inhibition (TD139) as well as knockout of Galectin-3 gene both attenuated Galectin-3–mediated suppression of regulatory T cells (T_reg_ cells) and protected from insulitis and diabetes onset in NOD mice. Mechanistically, Galectin-3 bound to and activated lymphocyte activation gene 3 (LAG3), a receptor expressed on activated T cells, subsequently suppressing the MEK/ERK signaling pathway and thereby hindering T_reg_ cell differentiation and function. In summary, our study identifies Galectin-3 as a potential biomarker for T1D and suggests that TD139 holds promise as a therapeutic candidate for patients with T1D and high serum Galectin-3 levels.

## Teaser

Galectin-3 activates LAG3 to inhibit T_reg_ cell differentiation and function, thereby triggering immune imbalance and insulitis.

## INTRODUCTION

Type 1 diabetes (T1D) is an autoimmune disease characterized by the progressive destruction of pancreatic β cells, resulting in absolute insulin deficiency (*1*). Regulatory T cell (T_reg_ cell) insufficiency is regarded as a critical driver of T1D. The progression of T1D in nonobese diabetic (NOD) mice is associated with a gradual loss of T_reg_ cell/effector T cell balance in the inflamed islets (*2*). Transcriptomic analysis revealed abnormalities in gene expression related to T_reg_ cell suppressive function in the pancreatic draining lymph nodes of NOD mice (*3*). Studies have demonstrated that depletion of T_reg_ cells markedly accelerates the development of T1D in NOD mice (*4*). Similarly, T_reg_ cells amplified ex vivo and adoptively transferred to NOD mice can control autoreactive T cells and halt disease development, which makes therapy with ex vivo expanded T_reg_ cells attractive (*5*).

The first clinical trial using polyclonal T_reg_ cell therapy for patients with T1D dates back to 2012, which showed that infusing ex vivo–expanded autologous CD4^+^CD127^lo^CD25^+^ polyclonal T_reg_ cells into newly diagnosed patients with T1D was well tolerated and significantly increased the percentage of circulating T_reg_ cells and improved plasma C-peptide levels (*6*). In addition, a 1-year follow-up study revealed that T_reg_ cell administration reduced the requirement for exogenous insulin (8 of 12 treated patients versus 2 of 10 untreated controls in remission) with two patients completely independent of insulin therapy at 1 year (*7*). Previous work of our team also showed that multiply restimulated human cord blood–derived T_reg_ cells maintained a stable phenotype and suppressive function, and the in vitro suppression rate of the T_reg_ cells cocultured with peripheral blood mononuclear cells (PBMCs) predicted their therapeutic effects on autoimmune diabetes (*8*). However, T_reg_ cell infusion still faces certain safety concerns, and the efficacy of each infusion can only be sustained for a limited period. Therefore, gaining a deeper understanding of the underlying causes and mechanisms behind the defects and dysfunction of T_reg_ cells in T1D is crucial for advancing the development of T_reg_ cell–based therapies.

Galectin-3, encoded by the *Lgals3* gene, is a member of the β-galactoside–binding lectin family (*9*, *10*). Recent studies have revealed that Galectin-3 plays a pivotal role in the pathogenesis of various autoimmune diseases by modulating immune cell functions and amplifying inflammatory responses, which collectively promote disease progression (*11*–*13*). Functional genomic studies combining proteomic and haplotype analyses have revealed that Galectin-3 polymorphisms are associated with T1D susceptibility (*14*). Subsequently, the role of Galectin-3 in T1D has been explored. Galectin-3–deficient (Galectin-3^−/−^) mice exhibit remarkable resistance to streptozotocin (STZ)–induced hyperglycemia development, accompanied by notable down-regulation of interferon-γ (IFN-γ), tumor necrosis factor–α, interleukin-17 (IL-17), and inducible nitric oxide synthase in macrophages (*15*). However, the role of extracellular (secreted) Galectin-3, as well as the alterations of circulating Galectin-3 levels in patients with T1D, remains unclear. Since Galectin-3 can be secreted into the extracellular environment, it exerts extracellular functions through its lectin activity, recognizing cell surface and extracellular matrix glycans to regulate cell surface receptor activities (*16*). Studies have demonstrated that extracellular Galectin-3 exerts multiple immunomodulatory effects; for instance, it can induce the migration and activation of monocytes and macrophages through Toll-like receptor 4 (TLR4) signaling (*11*, *17*). Moreover, Galectin-3 has been shown to affect the number and suppressive function of T_reg_ cells. In vitro studies revealed that treatment of PBMCs from healthy individuals with recombinant Galectin-3 down-regulated *FOXP3* mRNA expression (*18*). TD139 (also known as GB0139), a small-molecule inhibitor of Galectin-3 in phase 1/2 clinical trials for idiopathic pulmonary fibrosis (IPF) (NCT02257177) and COVID-19 pneumonitis (NCT04473053), exhibits high affinity for the carbohydrate recognition domain of Galectin-3, preferentially targeting extracellular Galectin-3, due to its limited cell permeability (*19*–*21*). TD139 treatment also increased the percentage of infiltrated T_reg_ cells and transforming growth factor–β (TGF-β)–producing T_reg_ cells among liver-infiltrating cells in an α-GalCer–induced hepatitis mouse model (*22*), suggesting that blockade of extracellular Galectin-3 promotes both T_reg_ cell expansion and function. However, the direct effect of Galectin-3 on T_reg_ cells, the key mediators of immune tolerance and autoimmune suppression, remains poorly understood in T1D.

In this study, we report that in patients with T1D, an elevated circulating lipopolysaccharide (LPS) level, resulting from increased intestinal permeability, stimulates monocytes/macrophages to secrete excessive Galectin-3, suggesting that Galectin-3 has the potential to serve as a promising biomarker for T1D. We found that Galectin-3 has pathological relevance in respect of in vivo T_reg_ cell deficiency in patients with T1D and NOD mice. We demonstrated that Galectin-3 impairs T_reg_ cell differentiation by binding to lymphocyte activation gene 3 (LAG3) and subsequently down-regulating MAPK kinase/extracellular signal–regulated kinase (MEK/ERK) signaling pathway, and the Galectin-3–specific inhibitor, TD139, can serve as a promising therapeutic agent for a T_reg_ cell defect, reducing insulitis and autoimmune diabetes. Together, our results identified that circulating Galectin-3 is a key mediator that subverts the suppressive function of T_reg_ cells. This mechanistic study has shed light on Galectin-3 as a potential therapeutic target for autoimmune diabetes.

## RESULTS

### Serum Galectin-3 is elevated in patients with T1D and their FDRs

**Fig. 1.**
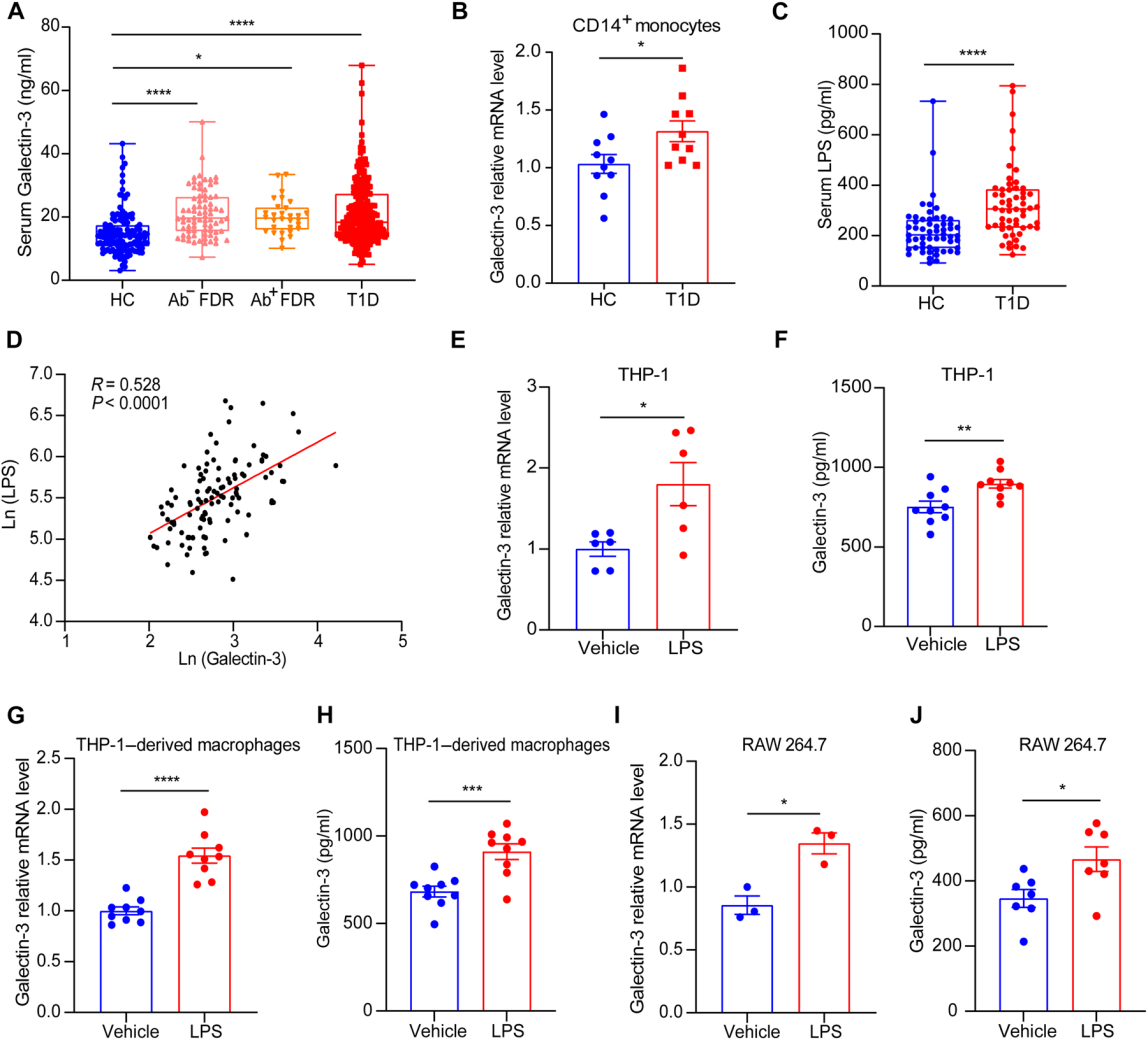
Serum Galectin-3 is increased in patients with T1D and their FDRs. (**A**) The concentration of fasting serum Galectin-3 in healthy controls (HC, *n* = 132), Ab^−^ FDR (*n* = 76), Ab^+^ FDR (*n* = 30), and patients with T1D (*n* = 234). Data are expressed as median ± range. (**B**) The mRNA expression levels of Galectin-3 in CD14^+^ monocytes isolated from patients with T1D and HC (*n* = 10), calculated using the 2^−△△Ct^ method. (**C**) The concentration of fasting serum LPS in HC and T1D groups (*n* = 56). Data are expressed as median ± range. (**D**) Correlation between serum levels of Galectin-3 and LPS in HC and patients with T1D. (**E** to **J**) The THP-1 cell line, THP-1–cell induced macrophages, and the RAW 264.7 cell line were treated with LPS (100 ng/ml) or vehicle for 24 hours. The mRNA expression levels of Galectin-3 [(E), (G), and (I)] and the protein concentration of Galectin-3 in the supernatant [(F), (H), and (J)] were measured. Data in (E) to (J) are representative of at least three independent experiments with similar results. Data are expressed as mean ± SEM. *P < 0.05, **P < 0.01, ***P < 0.001, and ****P < 0.0001.

To explore the clinical relevance of Galectin-3 in autoimmune diabetes, we first measured serum Galectin-3 levels in 234 patients with T1D and their first-degree relatives (FDRs) including 76 islet autoantibody–negative (Ab^−^ FDRs) and 30 islet autoantibody–positive (Ab^+^ FDRs), as well as 132 healthy controls (table S1). One-way analysis of variance (ANOVA) revealed a significant difference among groups comprising healthy controls, Ab^−^ FDRs, Ab^+^ FDRs, and patients with T1D (*P* < 0.0001). Circulating Galectin-3 levels were significantly elevated in patients with T1D compared to healthy controls (median 18.34 [IQR 14.21 to 27.23] versus 13.88 [IQR 10.84 to 17.34] ng/ml; *P* < 0.0001). Furthermore, compared with healthy controls, circulating Galectin-3 was increased in Ab^−^ FDRs (median 19.67 [IQR 15.61 to 26.23] versus 13.88 [IQR 10.84 to 17.34] ng/ml; *P* < 0.0001) and Ab^+^ FDRs (median 19.62 [IQR 16.12 to 22.89] versus 13.88 [IQR 10.84 to 17.34] ng/ml; *P* < 0.05), suggesting that augmented Galectin-3 production may occur before overt diabetes (Fig. 1A). Correlation analysis revealed that serum Galectin-3 levels were positively correlated with both fasting blood glucose (FBG) and glycated hemoglobin A1c (HbA1c) (table S2). Notably, Galectin-3 mRNA expression in peripheral CD14^+^ monocytes isolated from patients with T1D was significantly up-regulated compared to healthy controls (Fig. 1B), indicating that monocyte-derived Galectin-3 may contribute to the elevation of serum Galectin-3 in patients with T1D.

To further investigate the factors driving Galectin-3 up-regulation in circulating monocytes, we measured serum LPS, a well-established monocyte activator. We observed increased serum levels of LPS in patients with T1D (Fig. 1C) and a positive correlation between serum Galectin-3 and LPS levels (Fig. 1D), suggesting a potential mechanistic bridge between systemic endotoxemia and Galectin-3 overproduction. We then stimulated THP-1 cells, THP-1–derived macrophages, and RAW 264.7 cells with LPS. In all three cell types, LPS treatment robustly up-regulated Galectin-3 mRNA expression (Fig. 1, E, G, and I). In line with transcriptional up-regulation, LPS-stimulated THP-1 cells, THP-1–derived macrophages, and RAW 264.7 cells all showed significantly increased secretion of Galectin-3 protein into the conditioned media (Fig. 1, F, H, and J), indicating that endotoxemia observed in patients with T1D may promote Galectin-3 secretion by circulating monocytes. Given the established role of gut permeability in endotoxin translocation, we next assessed the intestinal barrier function of patients with T1D. Fresh ileum mucosal biopsies were obtained from five individuals with T1D and five age- and sex-matched healthy controls and subjected to single-cell RNA sequencing (scRNA-seq) (table S3). Analysis of the epithelial cell compartment revealed a marked reduction in the expression of key tight junction-associated genes, including *CLDN1* and *TJP1*, in patients with T1D compared to healthy controls (fig. S1, A and B), suggesting compromised epithelial barrier function in T1D. Galectin-3 mRNA levels in peripheral CD14^+^ monocytes from patients with T1D negatively correlated with the expression of gut barrier–associated genes (fig. S1, C and D). Together, these findings suggest that elevated serum Galectin-3 in T1D may result from increased intestinal permeability and subsequent LPS-driven activation of monocytes/macrophages, underscoring the potential of Galectin-3 to serve as a biomarker for preclinical autoimmune diabetes.

### Galectin-3 is predominantly expressed in islet macrophages at the early stage of autoimmune diabetes in NOD mice

**Fig. 2.**
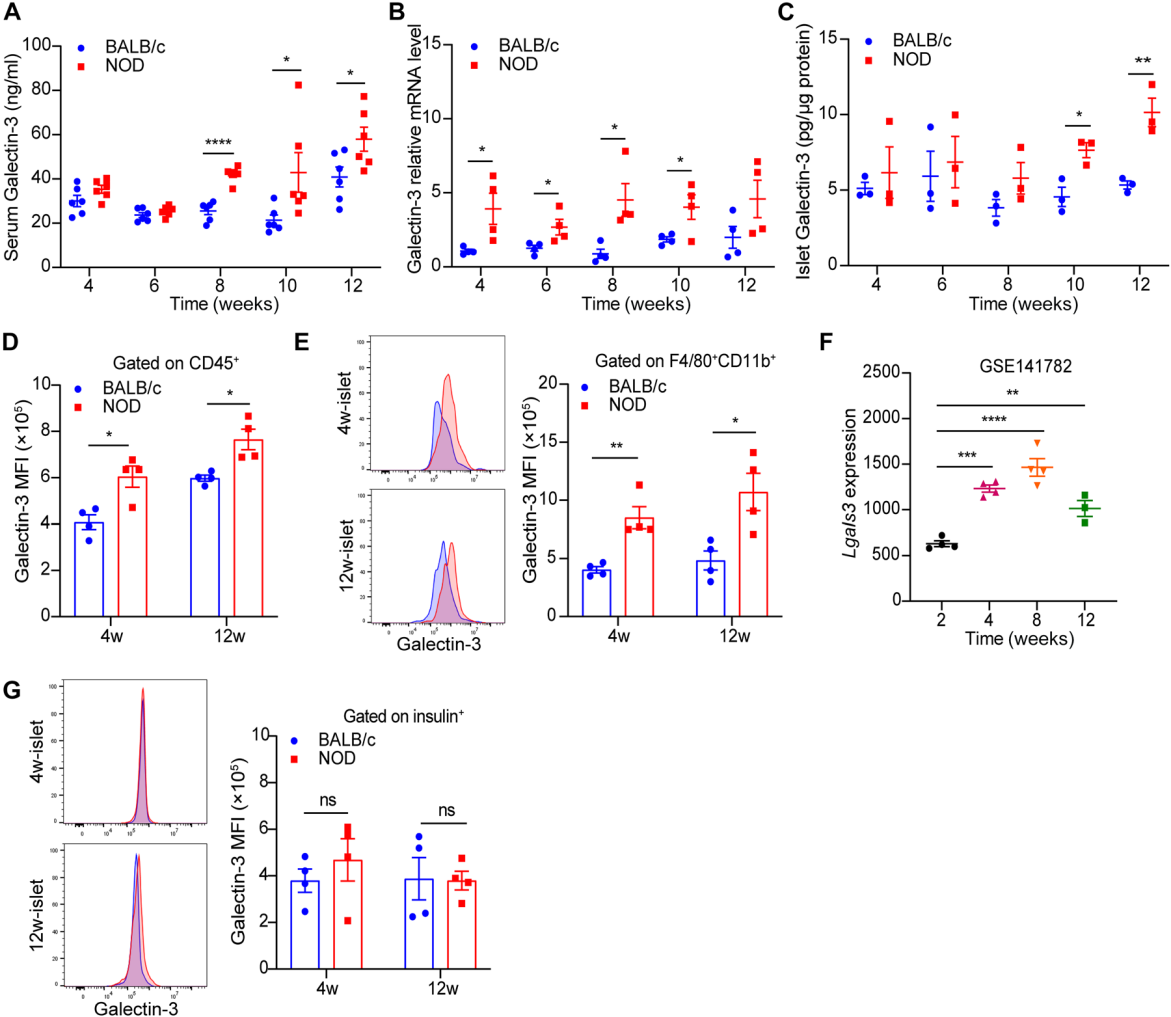
The expression of Galectin-3 in pancreatic islets is elevated at an early age in NOD mice. (**A**) Dynamic circulating levels of Galectin-3 in NOD and their control BALB/c mice at different ages (*n* = 6). (**B**) The mRNA abundance of Galectin-3 in the pancreatic islets of NOD and BALB/c mice, expressed as an arbitrary unit after normalization for β-actin mRNA levels, relative to the levels of 4-week-old BALB/c mice (*n* = 4). (**C**) The content of Galectin-3 in the pancreatic islet lysates of NOD and BALB/c mice (*n* = 3). (**D** and **E**) Representative patterns and median fluorescence intensity (MFI) of Galectin-3 expressed by CD45^+^ leukocytes (D) and CD45^+^F4/80^+^CD11b^+^ macrophages (E) in pancreatic islets of 4- and 12-week-old NOD and BALB/c mice (*n* = 4). (**F**) Galectin-3 expression in pancreatic islet macrophages of NOD mice at different ages (*n* = 3 to 4) from the National Center for Biotechnology Information database (GSE141782). (**G**) Representative patterns and MFI of Galectin-3 expressed by insulin^+^ pancreatic islet β cells of 4- and 12-week-old NOD and BALB/c mice (*n* = 4). Data are expressed as mean ± SEM. ns, no significance. *P < 0.05, **P < 0.01, ***P < 0.001, and ****P < 0.0001. 4w, 4 weeks; 12w, 12 weeks.

To further determine whether Galectin-3 plays a causal role in the pathogenesis of T1D, dynamic changes of Galectin-3 expression were investigated in the circulation and pancreatic islets of NOD mice at different ages. As insulitis progressed, NOD mice exhibited a significant increase in circulating Galectin-3 levels (Fig. 2A), accompanied by elevated Galectin-3 mRNA expression as well as protein abundance in pancreatic islets, compared to age-matched BALB/c controls (Fig. 2, B and C). Flow cytometry analysis further revealed that compared with BALB/c mice, CD45^+^ immunocytes, especially F4/80^+^CD11b^+^ macrophages, from pancreatic islets of 4- and 12-week-old NOD mice expressed higher Galectin-3 levels (Fig. 2, D and E). Analysis of bulk RNA-seq data from sorted islet macrophages of NOD mice from the public database (GSE141782) demonstrated that *Lgals3*, the gene encoding Galectin-3, was consistently significantly up-regulated at an early age in NOD mice before disease onset (Fig. 2F). In contrast, the expression levels of Galectin-3 in pancreatic β cells were comparable between NOD and BALB/c mice at either 4- or 12-week-old time points (Fig. 2G). Together, these results indicate that Galectin-3 is primarily up-regulated in islet macrophages during the early stages of autoimmune diabetes in NOD mice, implicating its potential involvement in insulitis and T1D pathogenesis.

### Galectin-3 deficiency alleviates autoimmune diabetes in NOD mice

**Fig. 3.**
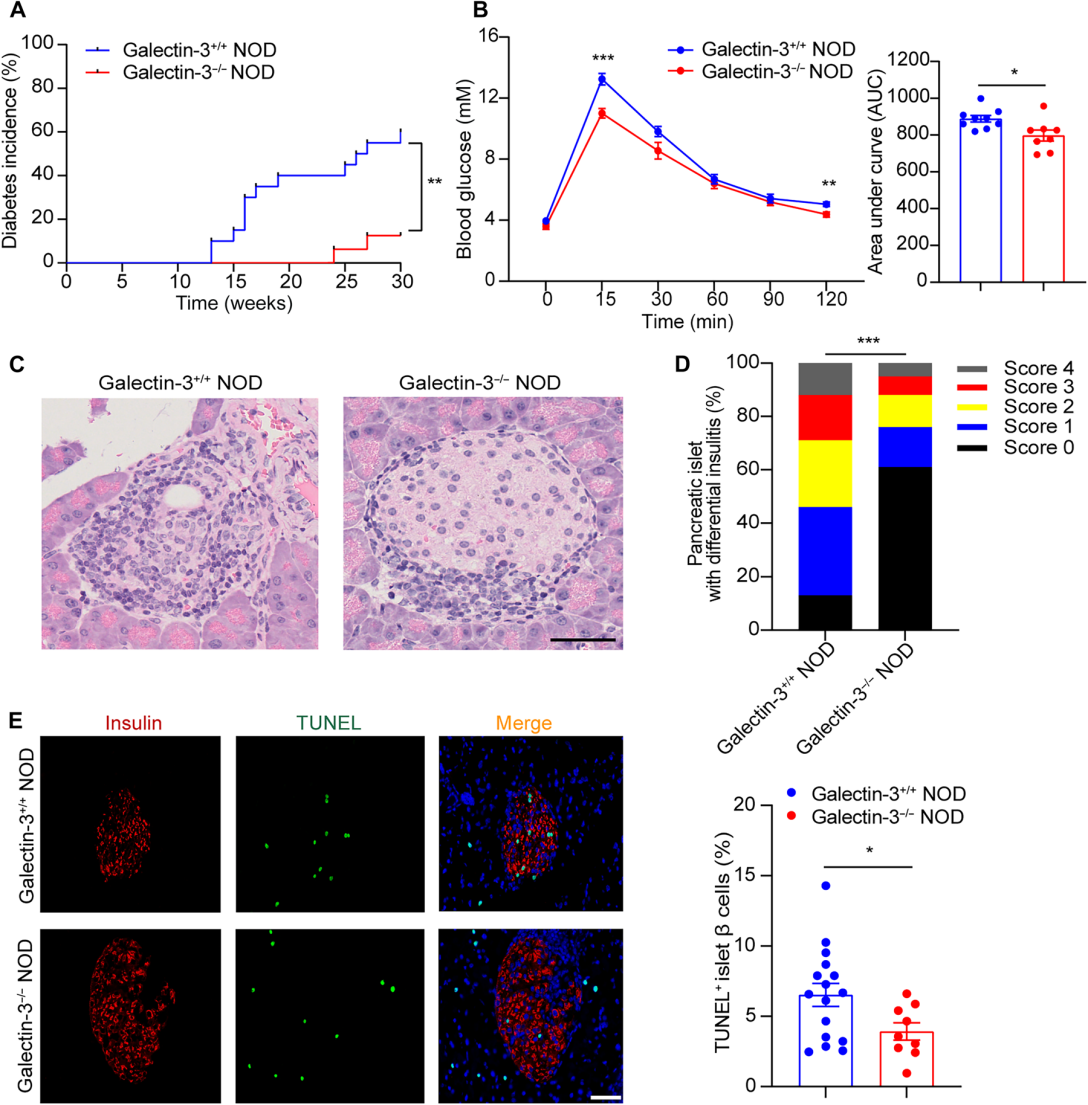
Galectin-3 deficiency alleviates autoimmune destruction of pancreatic islet β cells and diabetes in NOD mice. (**A**) Diabetes incidence in Galectin-3^+/+^ NOD (*n* = 20) and Galectin-3^−/−^ NOD mice (*n* = 16) at different ages. (**B**) Glucose excursion curve after receiving intraperitoneal glucose tolerance test (IPGTT) and the quantification of area under the curve in IPGTT (*n* = 8 to 9). (**C**) Representative images of H&E analysis for pancreatic sections and (**D**) insulitis scores in 12-week-old female Galectin-3^+/+^ NOD and Galectin-3^−/−^ NOD mice (*n* = 4). Scale bar, 50 μm. (**E**) Representative images of immunohistochemistry (IHC) staining of TUNEL (green) and insulin (red) in pancreas of 12-week-old female Galectin-3^+/+^ NOD and Galectin-3^−/−^ NOD mice (*n* = 6). Scale bar, 20 μm, with magnification of ×400. Data are expressed as mean ± SEM. *P < 0.05, **P < 0.01, and ***P < 0.001.

To address the role of Galectin-3 in T1D pathogenesis, we first used the CRISPR-Cas9 approach for gene editing of the *Lgals3* locus, thereby generating Galectin-3^−/−^ NOD mice (fig. S2A). We found that serum Galectin-3 levels of Galectin-3^−/−^ NOD were undetectable compared with Galectin-3^+/+^ NOD mice, confirming successful knockout of the *Lgals3* (fig. S2B). We next monitored the development of spontaneous diabetes in female Galectin-3^+/+^ NOD and Galectin-3^−/−^ NOD mice. Diabetes incidence was regularly monitored from 8 to 30 weeks of age. The onset of diabetes occurred as early as 13 weeks in Galectin-3^+/+^ NOD mice, whereas it was delayed to 24 weeks in Galectin-3^−/−^ NOD mice (Fig. 3A). At 30 weeks of age, diabetes incidence reached 60.0% in Galectin-3^+/+^ NOD mice but was significantly reduced to 12.5% in Galectin-3^−/−^ NOD mice (*P* < 0.01; Fig. 3A). Galectin-3^−/−^ NOD mice also displayed better glucose tolerance in response to the challenge of intraperitoneal glucose injection relative to Galectin-3^+/+^ NOD mice (Fig. 3B). Hematoxylin and eosin (H&E) staining analysis of pancreatic sections showed more severe islet destruction and much higher insulitis scores in 12-week-old Galectin-3^+/+^ NOD mice compared to Galectin-3^−/−^ NOD mice (Fig. 3, C and D). In situ cell death detection with terminal deoxynucleotidyl transferase–mediated deoxyuridine triphosphate nick end labeling (TUNEL) staining revealed that genetic ablation of Galectin-3 significantly reduced pancreatic β cell apoptosis in islets of NOD mice (Fig. 3E). Collectively, these findings support that Galectin-3 contributes to the development of insulitis, pancreatic β cell destruction, and autoimmune diabetes in NOD mice.

### Galectin-3 deficiency facilitates T_reg_ cell enrichment in pancreatic islets of NOD mice

**Fig. 4.**
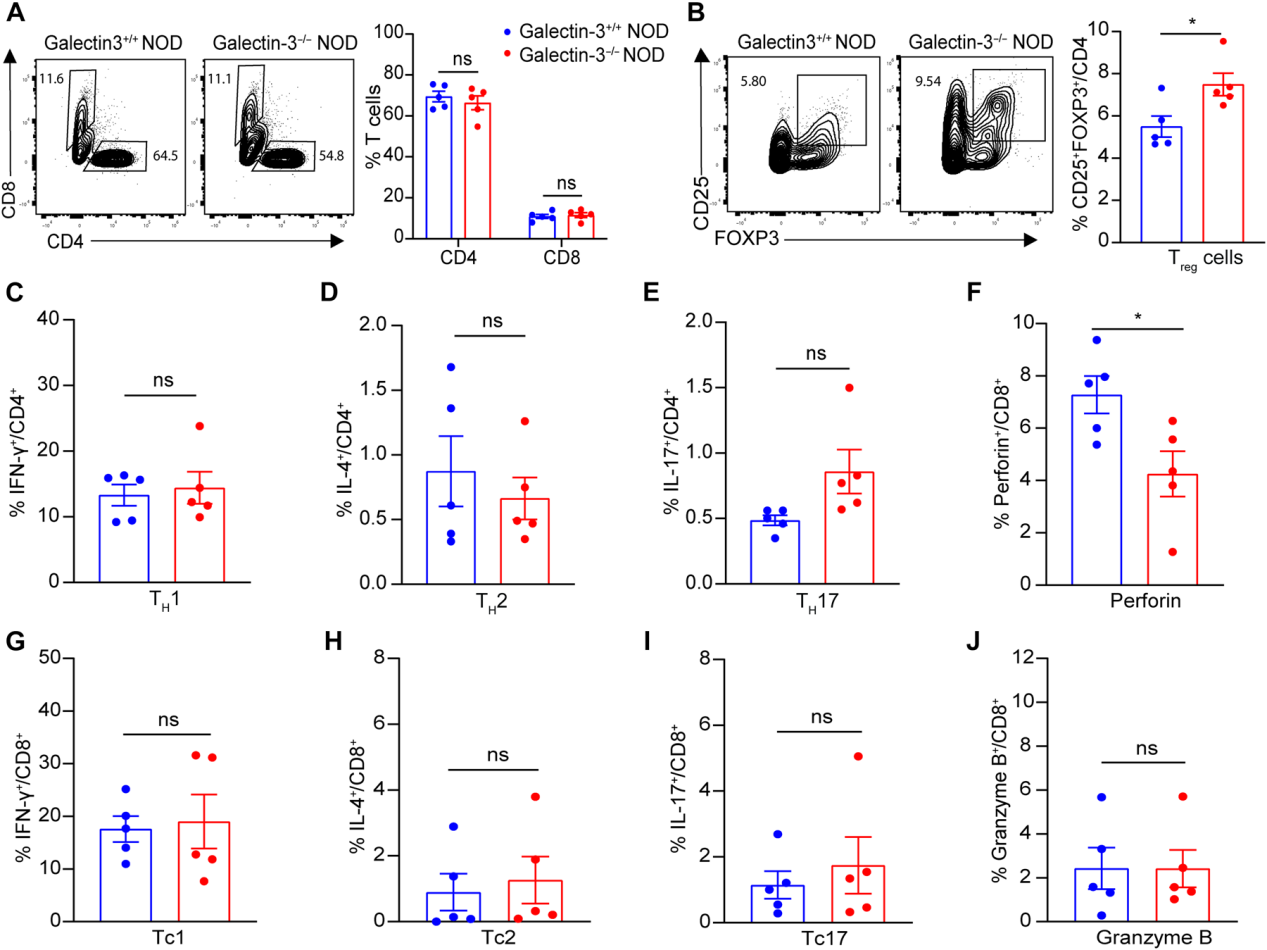
Galectin-3 deficiency enhances T_reg_ cell proportion and decreases perforin-expressing CD8^+^ T cells in pancreatic islets of NOD mice. Pancreatic islets from 12-week-old nondiabetic Galectin-3^+/+^ NOD and Galectin-3^−/−^ NOD mice were harvested and subject to flow cytometry analysis (*n* = 5). Frequencies of CD4^+^ and CD8^+^ T cells (**A**) and T_reg_ cells (CD4^+^CD25^+^FOXP3^+^) (**B**) are shown as representative fluorescence-activated cell sorting plots. (**C** to **E**) Frequencies of T_H_1 (CD4^+^IFN-γ^+^) (C), T_H_2 (CD4^+^IL-4^+^) (D), and T_H_17 (CD4^+^IL-17^+^) (E) subsets among CD45^+^ cells in islets of 12-week-old Galectin-3^+/+^ NOD and Galectin-3^−/−^ NOD mice. (**F** to **J**) Frequencies of perforin^+^CD8^+^ (F), Tc1 (CD8^+^IFN-γ^+^) (G), Tc2 (CD8^+^IL-4^+^) (H), Tc17 (CD8^+^IL-17^+^) (I), and granzyme B^+^CD8^+^ (J) subsets among CD45^+^ cells in islets of 12-week-old Galectin-3^+/+^ NOD and Galectin-3^−/−^ NOD mice. Data are expressed as mean ± SEM. *P < 0.05.

Given the indispensable role of T cell–mediated immunity in T1D pathogenesis, we next examined T lymphocyte profiling in the pancreatic islets of 12-week-old Galectin-3^+/+^ NOD and Galectin-3^−/−^ NOD mice using flow cytometry analysis. Although the proportions of CD4^+^ and CD8^+^ T cells were comparable between the two groups (Fig. 4A), Galectin-3^−/−^ NOD mice exhibited a significantly higher proportion of CD4^+^CD25^+^FOXP3^+^ T_reg_ cells (Fig. 4B). Regarding other CD4^+^ T cell subsets, there were no significant differences observed in the frequencies of T helper cell 1 (T_H_1; CD4^+^IFN-γ^+^), T_H_2 (CD4^+^IL-4^+^), or T_H_17 (CD4^+^IL-17^+^) cells between the two groups (Fig. 4, C to E). When further investigating the cytokine profile of CD8^+^ T lymphocytes, we found a significantly reduced proportion of perforin-expressing CD8^+^ T cells in Galectin-3^−/−^ NOD mice compared to Galectin-3^+/+^ controls (Fig. 4F). However, there were no significant differences in the frequencies of Tc1 (CD8^+^IFN-γ^+^), Tc2 (CD8^+^IL-4^+^), Tc17 (CD8^+^IL-17^+^) cells or granzyme B–producing CD8^+^ T cells between the two groups (Fig. 4, G to J). In addition, splenic T_reg_ cells were unchanged between Galectin-3^+/+^ NOD and Galectin-3^−/−^ NOD mice (fig. S3), indicating that the observed T_reg_ cell alterations are islet specific rather than systemic. Together, our data highlight that Galectin-3 deficiency promotes the expansion of T_reg_ cells and suppresses CD8^+^ T cell cytotoxicity in pancreatic islets, thereby reducing islet autoimmunity and ameliorating insulitis in NOD mice.

### Galectin-3 impairs T_reg_ cell differentiation and functionality

**Fig. 5.**
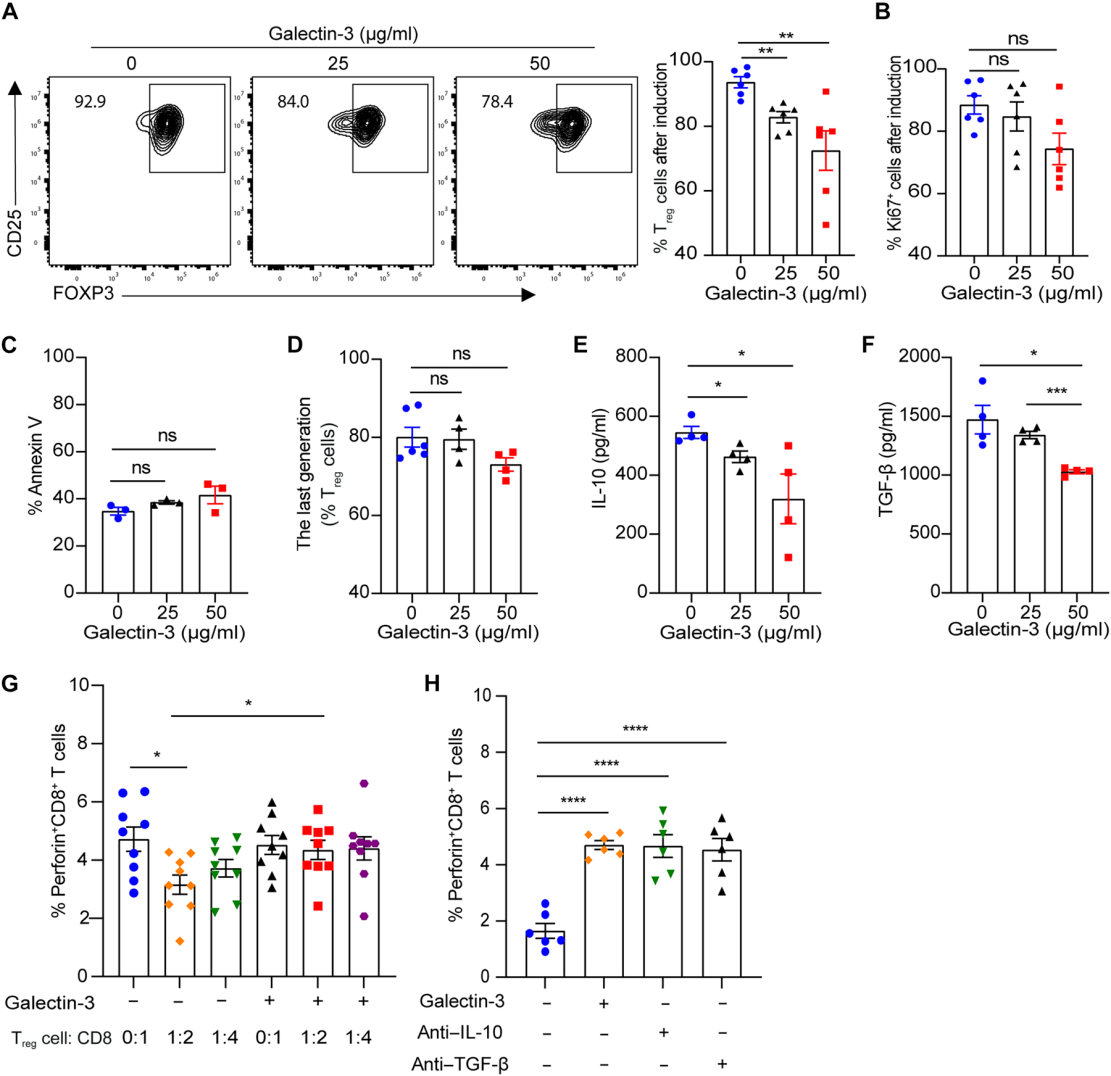
Galectin-3 exerts negative effects on T_reg_ cell differentiation and function. (**A**) Frequency of CD4^+^CD25^+^FOXP3^+^ T_reg_ cells after exposure of naïve CD4^+^ T cells to T_reg_ cell–inducing conditions with or without Galectin-3 recombinant protein at different concentrations (*n* = 6). (**B**) Frequency of Ki67^+^ cells after T_reg_ cell induction (*n* = 6). (**C**) Apoptosis of T_reg_ cells was determined by annexin V staining (*n* = 3). (**D**) The percentage of proliferated T_reg_ cells was defined by carboxyfluorescein diacetate succinimidyl ester assay after 72 hours of culture (*n* = 4 to 6). (**E** and **F**) Cytokine profile of culture supernatants of isolated T_reg_ cells (*n* = 4). (**G**) CD8^+^ T cells were stimulated with plate-coated anti-CD3/CD28 and cultured either alone or with Galectin-3–pretreated or vehicle-pretreated T_reg_ cells at the indicated ratios. Perforin expression in CD8^+^ T cells was measured by flow cytometry after 72 hours. (**H**) CD8^+^ T cells were cocultured with T_reg_ cells in a Transwell system in the presence or absence of Galectin-3 protein, IL-10, or TGF-β neutralizing antibodies. Perforin expression was measured by flow cytometry after 72 hours. Data are expressed as mean ± SEM. *P < 0.05, **P < 0.01, ***P < 0.001, and ****P < 0.0001.

To further investigate how Galectin-3 influences the frequency and function of T_reg_ cells in vivo, we first isolated naïve CD4^+^ T cells from wild-type (WT) C57BL/6J mice and activated them with anti-CD3/CD28 stimulation under T_reg_ cell–polarizing conditions. The addition of recombinant mouse Galectin-3 protein significantly suppressed the differentiation of naïve CD4^+^ T cells toward a T_reg_ cell fate without a perceptible impact on T cell proliferation (Fig. 5, A and B).

To assess the direct immunomodulatory effects of Galectin-3 on T_reg_ cells, we isolated CD4^+^CD25^+^ T_reg_ cells from splenocytes of WT C57BL/6J mice using magnetic-activated cell sorting (MACS). These MACS-purified T_reg_ cells were subsequently treated with recombinant mouse Galectin-3 protein, and we found that Galectin-3 had no significant effect on the apoptosis or proliferation of T_reg_ cells (Fig. 5, C and D). We then examined the effects of Galectin-3 on T_reg_ cell cytokine secretion. Galectin-3 treatment profoundly impaired the immunoregulatory capacity of T_reg_ cells through multiple mechanisms. T_reg_ cells exposed to Galectin-3 exhibited attenuated cytokine secretion including IL-10 (Fig. 5E) and TGF-β (Fig. 5F). In addition, Galectin-3–impaired T_reg_ cells also showed reduced expression of *Ctla4* and *Pdcd1*, key molecules mediating T_reg_–CD8^+^ T cell interactions (fig. S4). Furthermore, in direct coculture experiments, Galectin-3 pretreated T_reg_ cells failed to maintain suppressive capacity, resulting in markedly increased perforin expression by cocultured CD8^+^ T cells (Fig. 5G). An indirect coculture system by Transwell assay demonstrated that T_reg_ cell supernatants suppressed CD8^+^ T cell perforin production via soluble factors, as neutralizing antibodies against IL-10 and TGF-β restored perforin expression. Notably, this suppressive effect was abolished when T_reg_ cells were treated with Galectin-3 (Fig. 5H). Last, the pathological relevance of these findings was further corroborated in PBMCs from healthy donors which were cultured in vitro with recombinant human Galectin-3 protein. The administration of Galectin-3 consistently significantly decreased the frequency of CD4^+^CD25^+^FOXP3^+^ T_reg_ cells, while the proportions of T_H_1, T_H_2, and T_H_17 subsets remained unaffected (fig. S5). Collectively, our results demonstrate that Galectin-3 serves as a repressor of T_reg_ cell differentiation, cytokine secretion, and their ability to restrain CD8^+^ T cell cytotoxicity.

### Galectin-3 limits T_reg_ cell differentiation through LAG3-MEK/ERK signaling pathway

**Fig. 6.**
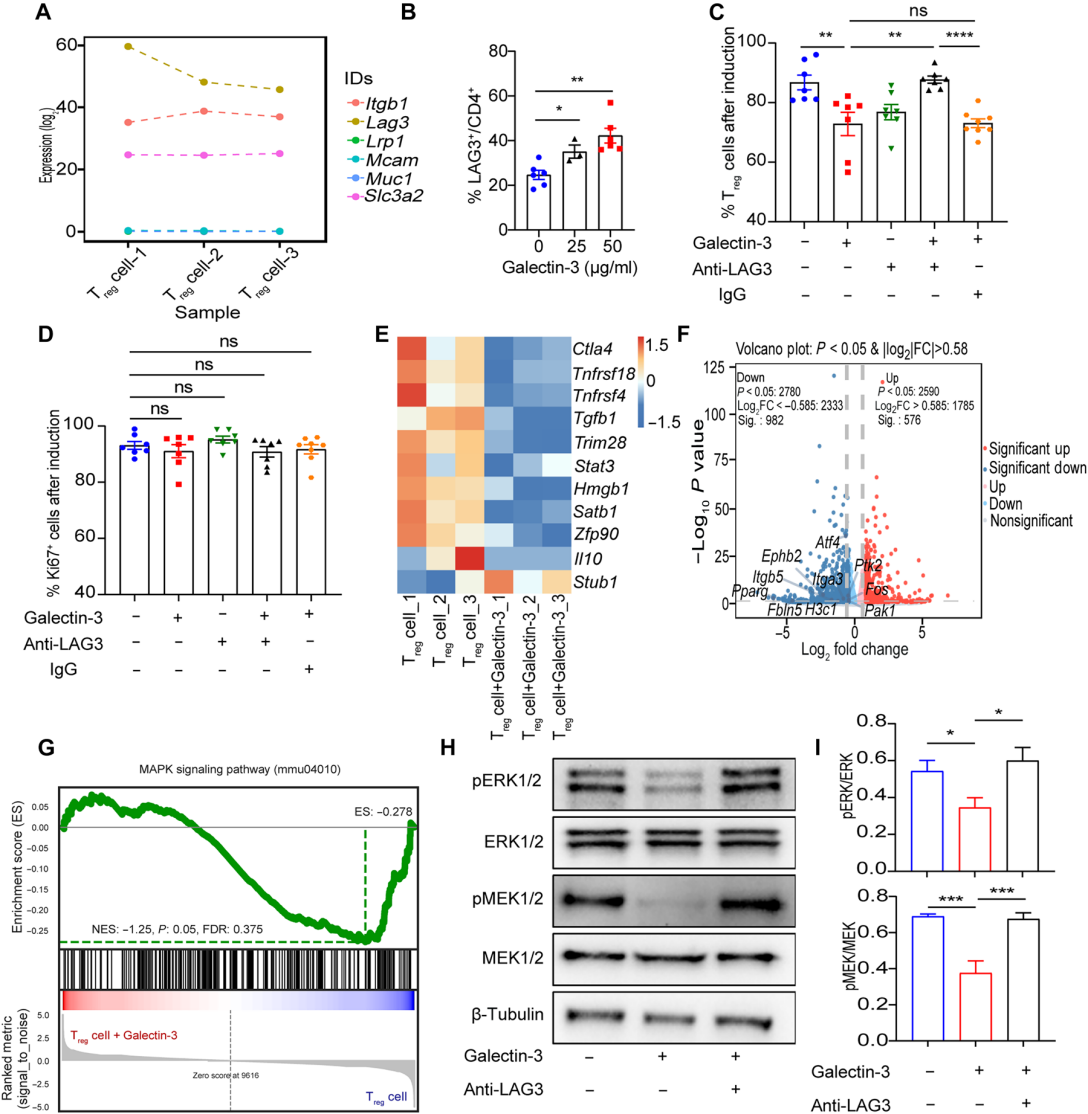
LAG3 signaling mediates the suppressive effects of Galectin-3 on T_reg_ cell differentiation. (**A**) Expression levels of candidate Galectin-3–binding receptors in T_reg_ cells (*n* = 3). (**B**) Expression of LAG3 was examined on T_reg_ cells after induction in different groups as indicated (*n* = 3 to 6). (**C** and **D**) Naïve CD4^+^ T cells purified from WT C57BL/6J splenocytes were cultured under T_reg_ cell–polarizing conditions in vitro, treated with Galectin-3 protein alone or in combination with LAG3 neutralizing antibody for 72 hours. The frequencies of CD4^+^CD25^+^FOXP3^+^ T_reg_ cells (C) as well as Ki67^+^ cells (D) were assessed by flow cytometry (*n* = 7). (**E**) Heatmap showing the expression of T_reg_ cell function–associated genes in control T_reg_ cells versus Galectin-3–treated T_reg_ cells (*n* = 3). (**F**) Volcano plot of DEGs between groups. Log_2_ FC, log_2_ fold change. (**G**) GSEA plot showing the enrichment of the MAPK signaling pathway in Galectin-3–treated T_reg_ cells compared to control T_reg_ cells. (**H** and **I**) Immunoblot analysis of MEK/ERK phosphorylation in T_reg_ cells stimulated with Galectin-3, with or without LAG3 blockade. Data are expressed as mean ± SEM. *P < 0.05, **P < 0.01, ***P < 0.001, and ****P < 0.0001. NES, normalized enrichment score; FDR, false discovery rate.

Our results in vitro thus far indicate that Galectin-3 directly impairs T_reg_ cell differentiation and function. Next, we sought to elucidate the mechanisms by which Galectin-3 impairs T_reg_ cell differentiation. We analyzed key Galectin-3 relevant receptors, such as LAG3 (*23*, *24*), CD98 (*25*), CD29 (*26*), MUC1 (*27*), LPR1 (*28*), and MCAM (*29*), and LAG3 emerged as the predominant receptor (Fig. 6A). Evidence has demonstrated that Galectin-3 directly interacts with LAG3, an immune checkpoint receptor typically considered to promote T_reg_ cell–mediated suppression; however, in autoimmune diabetes, LAG3 instead restricts T_reg_ cell proliferation and function (*30*, *31*). We found that Galectin-3 induced a significant increase in the expression of LAG3 on T_reg_ cells (Fig. 6B). Neutralization of LAG3 rescued the inhibition of T_reg_ cell differentiation induced by Galectin-3 (Fig. 6C) with a negligible effect on T_reg_ cell proliferation (Fig. 6D). To further elucidate the downstream mechanisms, we performed RNA-seq analysis to profile the transcriptomic landscape of Galectin-3–treated versus vehicle-treated T_reg_ cells. Galectin-3 treatment consistently down-regulated the expression of key genes associated with T_reg_ cell function, including *Ctla4*, *Tnfrsf18*, *Tnfrsf4*, *Tgfb1*, *Trim28*, *Stat3*, *Hmgb1*, *Satb1*, *Zfp90*, *Il10*, and up-regulated *Stub1*, which negatively regulates the suppressive function of T_reg_ cell (Fig. 6E). Galectin-3 treatment also significantly reduced expression of MEK/ERK pathway–related genes such as *Atf4*, *Ephb2*, *Fbln5*, *Fos*, *H3c1*, *Itga3*, *Itgb5*, *Ptk2*, *Pparg*, and *Pak1*, as confirmed by differentially expressed genes (DEGs) and gene set enrichment analysis (GSEA) (Fig. 6, F and G). Previous studies have reported that activation of the MAPK pathway, where MEK1/2 activates downstream ERK1/2 through phosphorylation, increases TGF-β and IL-2 production to promote T_reg_ cell differentiation (*32*). Western blot analysis further validated that Galectin-3 suppressed ERK1/2 and MEK1/2 phosphorylation, which was restored by LAG3 neutralizing antibody (Fig. 6, H and I). Collectively, these results indicate that Galectin-3 inhibits the MEK/ERK signaling in T_reg_ cells via interaction with LAG3, thereby impairing T_reg_ cell differentiation and functionality.

### Pharmacological inhibition of Galectin-3 prevents diabetes in NOD mice

**Fig. 7.**
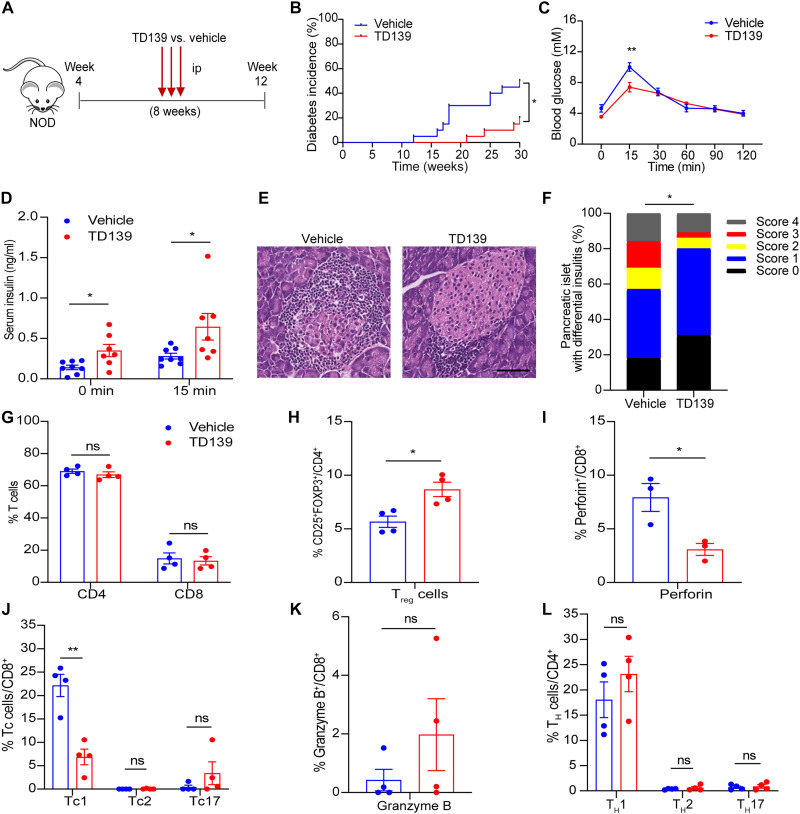
Pharmacological inhibition of Galectin-3 ameliorates spontaneous development of insulitis and diabetes in NOD mice. (**A**) Schematic diagram showing the protocol of TD139 treatment. (**B**) Incidence of diabetes expressed as percentage of diabetic mice at different ages (*n* = 20). (**C**) Glucose excursion curve after receiving IPGTT (*n* = 8). (**D**) Serum insulin concentration at 0 and 15 min during the IPGTT in 12-week-old TD139 or vehicle-treated NOD mice (*n* = 7 to 8). (**E**) Representative H&E staining of pancreas from 12-week-old NOD mice treated with TD139 or vehicle (*n* = 4). (**F**) Insulitis scores calculated on the basis of histological evaluation of pancreatic section as in (E). (**G**) Frequencies of CD4^+^ and CD8^+^ T cells in total CD3^+^ T cells from islets of 12-week-old NOD mice treated with TD139 or vehicle (*n* = 4). (**H**) Frequencies of T_reg_ cells (CD4^+^CD25^+^FOXP3^+^) from islets of 12-week-old NOD mice treated with TD139 or vehicle (*n* = 4). (**I** to **K**) Frequencies of perforin^+^CD8^+^ T, Tc1 (CD8^+^IFN-γ^+^), Tc2 (CD8^+^IL-4^+^), Tc17 (CD8^+^IL-17^+^), and granzyme B^+^CD8^+^ T subsets from islets of 12-week-old NOD mice treated with TD139 or vehicle (*n* = 3 to 4). (**L**) Frequencies of T_H_1 (CD4^+^IFN-γ^+^), T_H_2 (CD4^+^IL-4^+^), and T_H_17 (CD4^+^IL-17^+^) from islets of 12-week-old NOD mice treated with TD139 or vehicle (*n* = 4). Data are expressed as mean ± SEM. *P < 0.05 and **P < 0.01.

The pharmacological inhibitors of Galectin-3 have demonstrated efficacy in treating various diseases, including nonalcoholic steatohepatitis and pulmonary fibrosis in animal models, and even in humans (*20*, *33*–*35*). Considering our results that Galectin-3 knockout augmented the proportion of T_reg_ cells in islets and ameliorated insulitis in NOD mice, we sought to assess the potential therapeutic application of Galectin-3 inhibitors for treating T1D. Here, we evaluated the effect of TD139, a selective inhibitor with a high affinity for the Galectin-3 carbohydrate recognition domain in phase 2 clinical trials for IPF and COVID-19 pneumonitis (*20*, *21*), on spontaneous development of autoimmune diabetes in NOD mice. To this end, 4-week-old NOD mice were treated with TD139 (15 mg/kg per day) or vehicle by daily intraperitoneal injection for 8 weeks, followed by monitoring the incidence of diabetes until 30 weeks of age (Fig. 7A). Treatment with TD139 did not affect body weight or food intake (fig. S6). The onset of diabetes occurred as early as 12 weeks old in vehicle-treated NOD mice, while it was delayed to ~21 weeks old in TD139-treated NOD (Fig. 7B). At 30 weeks of age, ~50.0% of vehicle-treated NOD mice developed diabetes. In contrast, diabetes incidence of TD139-treated mice was significantly reduced to 20.0% (*P* < 0.05; Fig. 7B). To assess the impact of TD139 on pancreatic β cell function in 12-week-old NOD mice, we performed intraperitoneal glucose tolerance test (IPGTT) and found that pharmacological inhibition of Galectin-3 significantly improved glucose tolerance in NOD mice (Fig. 7C). TD139-treated mice consistently exhibited higher serum insulin levels at 0 and 15 min during the IPGTT compared to the vehicle-treated mice (Fig. 7D). H&E staining of the pancreatic sections and insulitis scoring analysis showed that TD139 treatment attenuated infiltration of immune cells and pancreatic β cell destruction (Fig. 7, E and F). Consistent with the phenotype observed in Galectin-3^−/−^ NOD mice, flow cytometry analysis showed that TD139 treatment increased T_reg_ cell frequency in the islets of NOD mice and reduced the perforin secretion capacity of islet CD8^+^ T cells, as well as Tc1 frequency (Fig. 7, G to J). No significant differences were found in other T cell subsets between the TD139-treated and vehicle-treated groups (Fig. 7, K and L). Similarly, TD139 did not increase splenic T_reg_ cells in NOD mice (fig. S7). Together, these data suggest that pharmacological inhibition of Galectin-3 from an early stage is sufficient to ameliorate pancreatic β cell destruction and diabetes in NOD mice.

## DISCUSSION

In this study, we demonstrated that serum Galectin-3 levels are significantly elevated in patients with T1D and even FDRs than in healthy controls and that serum Galectin-3 are positively correlated with circulating LPS concentrations resulting from increased intestinal permeability. Both genetic deletion of Galectin-3 and pharmacologic inhibition with TD139 rendered NOD mice resistant to insulitis and autoimmune diabetes. Mechanistically, our data suggest that extracellular Galectin-3 up-regulates LAG3 on T_reg_ cells and, in this context, limits their differentiation and suppressive capacity, thereby enhancing diabetogenic T cell activity. We therefore propose a model in which gut-derived low-grade endotoxemia drives myeloid secretion of Galectin-3, which in turn sustains constitutive LAG3 expression on T_reg_ cells and contributes to T_reg_ cell insufficiency and islet autoimmunity. Thus, these observations indicate that circulating Galectin-3 may serve as a promising biomarker for T1D risk stratification, and the Galectin-3 inhibitor may offer an immunoregulatory avenue for preventing T1D in high-risk individuals with elevated Galectin-3 levels.

Studies have shown that a disruption of the intestinal barrier function precedes the onset of T1D and causes bacterial endotoxin LPS translocation, and increased LPS in systemic circulation is causally linked to the inflammatory response (*36*). Circulating monocytes are key players in recognizing and responding to LPS through TLR4 activation (*37*). As an important proinflammatory cytokine which is mainly produced and secreted by monocytes/macrophages, Galectin-3 is up-regulated in both monocytes from patients with T1D and pancreatic islet macrophages from NOD mice. The mRNA expression of Galectin-3 in monocytes from patients with T1D was obviously correlated with gut permeability, determined by *CLDN1* and *TJP1* levels, which are major modulators of intercellular tight junction and intestinal mucus, and serum Galectin-3 levels were related significantly to serum LPS levels. Of note, Galectin-3 production could be significantly increased after LPS stimulation in monocyte-like THP-1, THP-1–induced macrophages, and RAW 264.7 cells. Thus, we speculate that the LPS-induced monocyte/macrophage inflammation is mainly responsible for the elevated circulating Galectin-3 levels in patients with T1D and individuals at high risk for T1D, which could be regarded as a promising clinical biomarker for T1D.

Notably, Galectin-3 exerts diverse functions depending on its intracellular or extracellular localization. Transgenic overexpression of Galectin-3 in pancreatic β cells has been shown to attenuate hyperglycemia in STZ-induced diabetic mice, suggesting its antiapoptotic role within pancreatic β cells (*38*). However, we found no difference in Galectin-3 expression within pancreatic β cells of NOD mice compared with controls, nor between 4-week (peri-insulitis) and 12-week (evident insulitis) NOD mice, indicating that during chronic autoimmune destruction, as opposed to acute toxin-induced injury, pancreatic β cell–intrinsic Galectin-3 is not compensatorily increased to exert protective effects. Recent evidence also showed that extracellular Galectin-3 impaired glucose-stimulated insulin secretion in pancreatic β cells in type 2 diabetes (T2D) mouse models (*39*). However, the metabolic stress on pancreatic β cells, driven by glucotoxicity and lipotoxicity in T2D, differs fundamentally from the immunopathogenesis of T1D. Although we cannot exclude the possible impact of Galectin-3 on pancreatic β cells in T1D, the immune modulatory effects appear to be dominant in our experimental setting by using a NOD mouse model. Our in vitro experiments demonstrate that extracellular Galectin-3 critically disrupts T_reg_ cell differentiation and function and subsequently enhances CD8^+^ T cell cytotoxicity.

In autoimmune diseases, Galectin-3 often functions as a proinflammatory amplifier, including in experimental autoimmune encephalomyelitis and rheumatoid arthritis (*40*, *41*). Our study found that serum Galectin-3 was markedly elevated in both patients with T1D and NOD mice, together with the observation that Galectin-3–deficient and WT naïve T cells generated comparable CD4^+^CD25^+^FOXP3^+^-induced T_reg_ cells under in vitro polarization (fig. S8), indicating that extracellular Galectin-3 engaging T cells, rather than intracellular Galectin-3 within T cells, plays the primary role in T1D. Studies have shown that extracellular Galectin-3 regulates a range of biological processes in T cells, including activation, proliferation, apoptosis, cytokine secretion, and signaling (*16*, *42*). Evidence from T cell lines indicates that the addition of Galectin-3 in vitro can induce cytosolic Ca^2+^ signaling to promote T cell activation (*43*) and directly induce T cell apoptosis in a concentration-dependent manner (*44*). Recent studies have highlighted the significance of Galectin-3 in cancer as an intrinsic tumor escape mechanism. Specifically, Galectin-3 reduces IFN-γ diffusion through the tumor matrix avoiding the production of an IFN-γ–induced chemokine (such as CXCL9) required for T cell recruitment (*45*) and interacts with Galectin-3 binding protein from extracellular vesicles to suppress the activation of CD4, CD8, and CD56 effector cells through CD45 receptor, further promoting tumor escape (*46*). In addition, Galectin-3 released by pancreatic ductal adenocarcinoma cells after coculture with γδ T cells binds glycosylated γδ T cell surface receptor α3β1 integrin and contributes to inhibition of γδ T cell proliferation (*47*). In contrast, in an α-GalCer–induced hepatitis mouse model, pharmacological Galectin-3 neutralization could increase the percentage of CD4^+^CD25^+^FOXP3^+^ T_reg_ cells and TGF-β–producing T_reg_ cells among liver-infiltrating cells (*22*). In our study, both genetic knockout and pharmacologic inhibition of Galectin-3 increased islet T_reg_ cell frequencies and reduced perforin-expressing CD8^+^ T cells, whereas other T cell subsets were unchanged. Thus, the mechanism of Galectin-3–mediated T cell suppression may be disease specific.

LAG3 (also named CD223), an immune checkpoint receptor, is expressed on the surface of activated effector T cells and T_reg_ cells (*48*, *49*). Previous studies have shown that Galectin-3 suppresses T cell activation via binding to LAG3. In pancreatic ductal adenocarcinoma, Galectin-3 was shown to engage CD8^+^ T cells coexpressing PD-1 and LAG3 in the tumor microenvironment, although coimmunoprecipitation experiments confirmed binding to LAG3 but not PD-1 (*24*). Functionally, Galectin-3 depletion restored T cell–mediated tumor control (*42*). However, this mechanism of Galectin-3–mediated T cell suppression may be disease specific. In chronic viral infection, Galectin-3–induced T cell suppression did not involve either PD-1 or LAG3 (*50*). Another study also demonstrated that immune checkpoint blockade could mitigate Galectin-3–dependent T cell dysfunction (*51*). In melanoma, CTLA-4 and vascular endothelial growth factor A blockade elicited a humoral response to Galectin-3 that correlated with improved patient outcomes (*51*). Accumulating evidence underscores the pivotal role of the MEK/ERK signaling cascade in T_reg_ cell differentiation and functional competence. ERK phosphorylation is indispensable for TGF-β–induced FOXP3 expression during T_reg_ cell differentiation (*52*). Further, sustained MEK activity potentiates T_reg_ cell–mediated immunosuppression by up-regulating key functional markers including CTLA-4 and GITR through epigenetic remodeling of their promoter regions (*53*, *54*). In our study, we expand on these findings by demonstrating that Galectin-3 interacts with LAG3 on CD4^+^ T cells and inhibits T_reg_ cell differentiation through suppression of the MEK/ERK signaling. Notably, blockade of LAG3 reversed the inhibitory effects of Galectin-3 on T_reg_ cells. Overall, the Galectin-3–LAG3 axis plays a disease-contextual role in modulating T cell immunity and may represent a promising therapeutic target in autoimmune diabetes.

Although our study provides substantial evidence that Galectin-3 disrupts T_reg_ cell–mediated immune tolerance and thereby promotes progression toward T1D, several limitations should be noted. First, while elevated circulating Galectin-3 in Ab^−^ FDRs may reflect subclinical immune dysregulation and suggest underlying increased seroconversion or T1D risk, our human data are limited by the cross-sectional design. Large-scale prospective clinical cohorts are needed to establish the independent predictive value of Galectin-3 for T1D risk stratification. Second, because of the inherent model constraint, further validation in myeloid-derived Galectin-3 deletion or overexpression models is warranted. Third, given that the biological effects of extracellular Galectin-3 remain relatively extensive, the application of TD139 may be limited by potential off-tissue effects. To further address this challenge, therapeutic agents targeted delivery to pancreatic islets could maximize the therapeutic efficacy in T1D while circumventing systemic immunosuppression.

In conclusion, Galectin-3, which is increased in the serum of patients with T1D, limits the production and function of T_reg_ cells and therefore exacerbates islet autoimmunity and islet β function failure. Our study also proposes the activation of LAG3, for weakening the differentiation of T_reg_ cells, which may account for insufficient T_reg_ cells in NOD mice or patients with T1D with elevated circulating Galectin-3 levels. Our findings suggest a promising avenue for potential therapeutic targets of T1D and introduce the Galectin-3 inhibitor TD139, as a potent antidiabetic drug, to prevent and treat T1D.

## MATERIALS AND METHODS

### Human study

All participants were recruited from the Second Xiangya Hospital of Central South University, Changsha, China. A total of 234 patients with T1D and 106 FDRs defined as at least one patient with T1D among parents, siblings, or children (including Ab^−^ and Ab^+^ FDRs) of patients with T1D were recruited for this study. All patients enrolled in the present study fulfilled the 1999 World Health Organization criteria for diabetes (*55*). Patients with T1D were diagnosed according to the criteria of the American Diabetes Association (*56*). In addition to the diagnosis of diabetes, the following were also considered in the diagnosis of T1D: symptoms, signs, history, dependence of insulin application, absence of pancreatic β cell function, and were positive for at least one of islet autoantibodies, including glutamic acid decarboxylase antibody (GADA), insulinoma-associated protein 2 antibody (IA-2A), or zinc transporter-8 antibody (ZnT8A). Newly diagnosed T1D was defined as clinical diabetes with a disease duration of within 3 months from diagnosis as described before (*57*). All FDRs of patients with T1D had normal glucose tolerance (NGT) with FBG of less than 5.6 mM and 2-hour blood glucose of less than 7.8 mM by oral glucose tolerance test (OGTT) and were screened for islet autoantibodies (GADA, ZnT8A, and IA-2A). Ab^+^ FDRs were defined as individuals positive for at least one autoantibody against GADA, IA-2A, or ZnT8A. A group of 132 healthy controls was recruited with NGT confirmed by OGTT and with no family history of diabetes or other autoimmune diseases.

All human sample study protocols were approved by the Institutional Review Board of The Second Xiangya Hospital of Central South University (no. 2022-262). All of the study participants provided informed consent.

### Clinical and biochemical assessments

For all participants, height, weight, and hip circumference were measured with a standardized procedure. Body mass index was calculated on the basis of those measurements.

Venous blood samples were drawn in the fasting state and 2 hours after a meal. After 2 hours at room temperature, serum samples were centrifuged at 1500*g* for 10 min. Blood glucose level was analyzed by a Hitachi 7170 analyzer (Boehringer, Mannheim, Germany). Serum cholesterol, triglycerides, high-density lipoprotein cholesterol, and low-density lipoprotein cholesterol levels were measured by enzymatic assays. Serum levels of C-peptide were measured using the Advia Centaur System (Siemens, Munich, Germany). HbA1c was detected by ion exchange high-performance liquid chromatography (HLC-723G8, Tosoh, Japan), and intraassay and interassay differences were <1 and <3%, respectively. GADA, IA-2A, and ZnT8A autoantibodies were detected by the radioligand assay with in vitro–translated 35*S* methionine–labeled GAD65, IA-2, or ZnT8 as we described previously (*58*). The fasting serum Galectin-3 concentration of the subjects was measured by a quantitative enzyme-linked immunosorbent assay (ELISA) kit (DY1197, R&D, Minneapolis, USA). Serum LPS was determined using an ELISA kit (CSB-E09945h, Cusabio, Wuhan, China) according to the manufacturer’s instructions. All samples were tested in duplicate, and the average value was used for data analysis.

### scRNA-seq of human intestinal tissue

Fresh ileum samples were obtained from colonoscopy biopsies and immediately processed for scRNA-seq. The scRNA-seq protocol involved the following steps: tissue dissociation, cell sorting, library preparation, and sequencing. The resulting sequencing data were analyzed using Seurat to identify distinct cell clusters and determine their gene expression profiles.

### Isolation of human monocytes

PBMCs from healthy controls and patients with T1D were isolated from the heparinized whole blood by density gradient centrifugation with Histopaque 1077 (Sigma-Aldrich, USA). Subsequently, cells were washed with phosphate-buffered saline (PBS), followed by isolation of monocytes from PBMCs using anti-human CD14 immunomagnetic beads (Miltenyi Biotec, Germany). Cells were harvested, and RNA was extracted with the Total RNA Extraction Kit (Promega, China) and reverse transcribed into cDNA (TransGen, China). Real-time polymerase chain reaction (PCR) was performed using quantitative PCR master mix (Promega, China) on the QuantStudio 6 Flex System (Applied Biosystems, USA).

### Animals

NOD/ShiLtJ (namely NOD), BALB/c, and C57BL/6J mice were purchased from Beijing HFK Bioscience (Beijing, China). Galectin-3^−/−^ mice in the NOD background provided by Gempharmatech Co. Ltd. were generated using CRISPR-Cas9 gene editing. Genotypes of these mice were confirmed using primers in table S3. Age-matched female Galectin-3^+/+^ NOD mice and Galectin-3^−/−^ NOD mice were used in the experiments of this study. All of the mice were housed in specific pathogen–free conditions with temperature (22° ± 1°C), humidity (50 to 70%), and light (12-hour light/dark cycle) control and were housed in individually ventilated cages with free access to water and food unless otherwise indicated. After 8 weeks of age, female NOD mice were monitored for blood glucose twice per week using a blood glucose test meter (ACCU-CHEK) and strips (Roche Diagnostics, USA) and diagnosed as diabetic once two consecutive blood glucose readings were >13.9 mM. All animal studies and protocols were approved by the Animal Care and Use Committee of the Second Xiangya Hospital of Central South University (no. 2022062).

### Isolation of pancreatic islets

Pancreas from age-matched Galectin-3^+/+^ NOD mice and Galectin-3^−/−^ NOD mice were perfused with 3 ml of a solution containing collagenase V (1 mg/ml; Sigma-Aldrich) and then digested for 15 min at 37°C. Digestion was stopped by adding D-Hank’s [containing 5% fetal bovine serum (FBS)] followed by extensive washes. After centrifugation at 300*g* for 2 min, the islets were resuspended and cultured in RPMI 1640 medium with 10% FBS (Gibco, USA) and 1% penicillin and streptomycin (Gibco, USA) and further purified and counted by hand picking under a microscope (Bx41 System, Olympus).

### Intraperitoneal glucose tolerance test

Mice housed in clean cages were fasted for 16 hours before intraperitoneal injection with d-glucose (1 g/kg). Venous blood glucose was measured at 0, 15, 30, 60, 90, and 120 min after glucose challenge to measure glucose levels with a blood glucose test meter (ACCU-CHEK) and strips (Roche Diagnostics, USA) according to the manufacturer’s instructions.

### Immunostaining

For immunofluorescence staining, antigen retrieval was first performed in boiled sodium citrate buffer and then blocked for 1 hour. The sections were probed with primary antibodies against insulin and incubated overnight at 4°C in a wet box, followed by sequential incubation with secondary antibodies at room temperature for 30 min.

### Flow cytometry analysis

Single-cell suspensions were prepared from various tissues of the mice in different groups. Cells were then harvested by centrifugation at 300*g* for 10 min at 4°C, resuspended in 100 μl of Live/Dead Fixable Dead Cell Stains (BD Biosciences, USA), incubated on ice for 10 min, and then washed with 1× PBS, followed by staining with different antibodies. For intracellular cytokine assay, cells were stimulated with cell stimulation cocktail (Invitrogen, USA) in cell culture medium at a density of 2 × 10^6^ cells/ml for 6 hours at 37°C. An Fc receptor blocker was added to block nonspecific binding. Surface markers were added before fixation and permeabilization, and intracellular cytokine staining was then performed according to a protocol. Cells were analyzed using the NovoCyte Quanteon 4025 Flow Cytometry System (Agilent, USA), and data analysis was performed on FlowJo software version X.0.7.

### Cell purification and polarization

Naïve T cells were enriched with a Naïve CD4^+^ T Cell Isolation Kit for a mouse (130-104-453, Miltenyi Biotec), and T_reg_ cells were enriched with a CD4^+^CD25^+^ Regulatory T Cell Isolation Kit for mouse (130-091-041, Miltenyi Biotec) according to the manufacturer’s instructions. The sorted T cells were cultured in RPMI 1640 medium (plus β-mercaptoethanol) supplemented with 10% FBS, 1% GlutaMAX, 1% sodium pyruvate, and 1% penicillin and streptomycin (all from Gibco) for further study. For T_reg_ cell polarization, naïve CD4^+^ T cells were activated with plate-coated anti-CD3 (5 μg/ml) and anti-CD28 (BD Biosciences, USA) for 72 hours in the presence of IL-2 (10 ng/ml; R&D, USA) and TGF-β (5 ng/ml; PeproTech, USA). For LAG3 blockade experiments, cells were treated with either anti-LAG3 antibody (10 μg/ml; Bio X Cell, BE0174) or an equivalent concentration of immunoglobulin G (IgG) isotype control (rat IgG2b, κ; Bio X Cell, BE0090).

### T cell proliferation assay

CD4^+^CD25^+^ T_reg_ cells purified from the spleens of WT C57BL/6J mice were stained with carboxyfluorescein succinimidyl ester (BioLegend, USA). The cells (1 × 10^6^/ml) were plated in the anti-CD3– and anti-CD28–coated 96-well plate. Cell proliferation was evaluated after 72 hours by detecting CFSE dye dilution using flow cytometry.

### RNA isolation and library preparation

Total RNA was extracted using the TRIzol reagent (Invitrogen, CA, USA) according to the manufacturer’s protocol. RNA purity and quantification were evaluated using the NanoDrop 2000 spectrophotometer (Thermo Fisher Scientific, USA). RNA integrity was assessed using the Agilent 2100 Bioanalyzer (Agilent Technologies, Santa Clara, CA, USA). Then, the libraries were constructed using the VAHTS Universal V6 RNA-seq Library Prep Kit according to the manufacturer’s instructions. The transcriptome sequencing and analysis were conducted by OE Biotech Co. Ltd. (Shanghai, China).

### RNA-seq and DEG analysis

The libraries were sequenced on an Illumina NovaSeq 6000 platform, and 150-bp paired-end reads were generated. About 46.37 million raw reads for each sample were generated. Raw reads of fastq format were first processed using fastp, and the low-quality reads were removed to obtain the clean reads. Then, about 45.31 million clean reads for each sample were retained for subsequent analyses. The clean reads were mapped to the reference genome using HISAT2. FPKM of each gene was calculated, and the read counts of each gene were obtained by HTSeq-count. Principal components analysis was performed using R (v3.2.0) to evaluate the biological duplication of samples.

Differential expression analysis was performed using the DESeq2. *P* < 0.05 and fold change > 1.5 or fold change < 0.667 were set as the threshold for DEGs. Hierarchical cluster analysis of DEGs was performed using R (v3.2.0) to demonstrate the expression pattern of genes in different groups and samples. The radar map of top 30 genes was drawn to show the expression of up-regulated or down-regulated DEGs using R package ggradar.

GSEA was performed using the GSEA software, with genes ranked by their degree of differential expression between the two sample types based on a predefined gene set. The analysis tested whether the predefined gene set was significantly enriched at the top or bottom of the ranked list.

### Real-time PCR and immunoblot analysis

Real-time PCR and immunoblot analysis were performed as previously reported (*59*). Primer sequences for all examined genes are listed in table S5.

### Statistical analysis

Data are presented as the mean ± SEM for normally distributed variables or as the median (25th to 75th percentiles) for nonnormally distributed variables. All in vitro studies were performed at least three times, and results are presented as mean ± SEM. Statistical analysis and data visualization were performed using SPSS 26.0 and GraphPad Prism 9, respectively. Correlation analyses were conducted using Spearman’s rank correlation. Diabetes incidence was compared using the log-rank (Mantel-Cox) test. IPGTT results were analyzed with one-way ANOVA, the insulitis score was analyzed by the chi-square test, and the remaining data were analyzed using the Student’s *t* test, unless otherwise stated. All statistical tests with *P* < 0.05 were considered significant.
