# Supplementary material for: Galectin-3 exacerbates autoimmune diabetes by limiting regulatory T cell differentiation and function
Source: Sci Adv. 2026 Jan 1;12(1):eadz7916. doi: 10.1126/sciadv.adz7916 (PMC12757048; doi:10.1126/sciadv.adz7916)
Supplement: Supplementary file 1 — Figs. S1 to S8 Tables S1 to S6 [file sciadv.adz7916_sm.pdf]

Science Advances
AAAS

# Supplementary Materials for

## Galectin-3 exacerbates autoimmune diabetes by limiting regulatory T cell differentiation and function

Lingxiang Xie *et al.*

Corresponding author: Yang Xiao, xiaoyang29@csu.edu.cn

*Sci. Adv.* **12**, eadz7916 (2026)
DOI: 10.1126/sciadv.adz7916

**This PDF file includes:**

Figs. S1 to S8
Tables S1 to S6

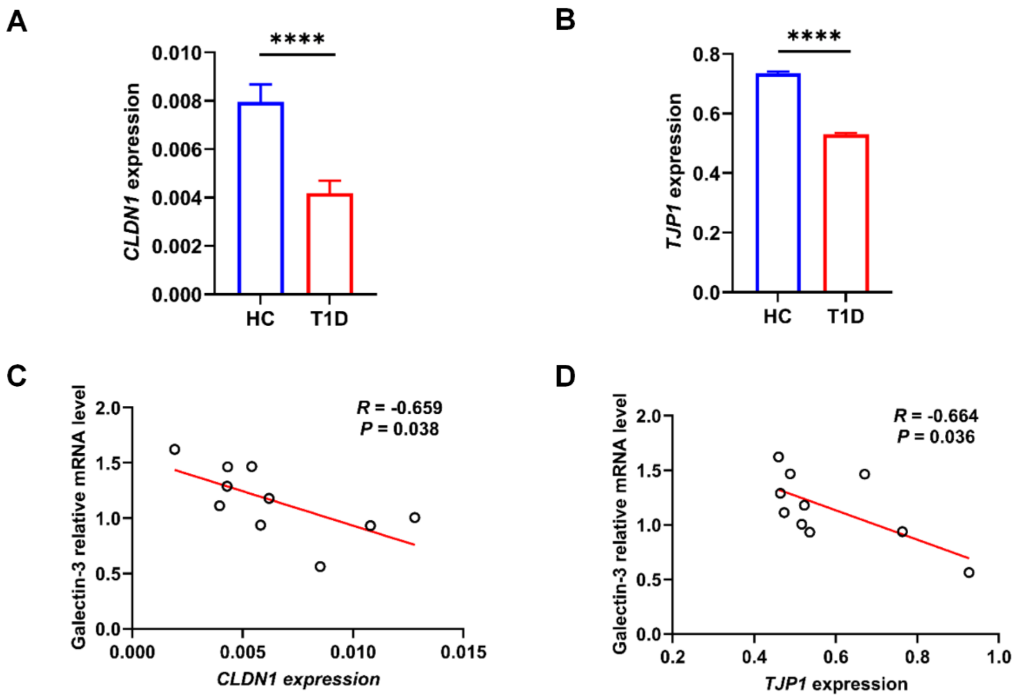


**Fig. S1. T1D patients exhibit impaired gut barrier function.** (**A** and **B**) Expression of gut barrier-related markers *CLDN1* (A) and *TJP1* (B) in intestinal epithelial cells from HC and T1D participants (n = 5 per group), as analyzed by single-cell RNA sequencing (scRNA-seq). (**C** and **D**) Correlation between Galectin-3 mRNA levels in peripheral CD14$^{+}$ monocytes and gut barrier-related genes expression. Data are expressed as mean ± SEM. ****$P$ < 0.0001.

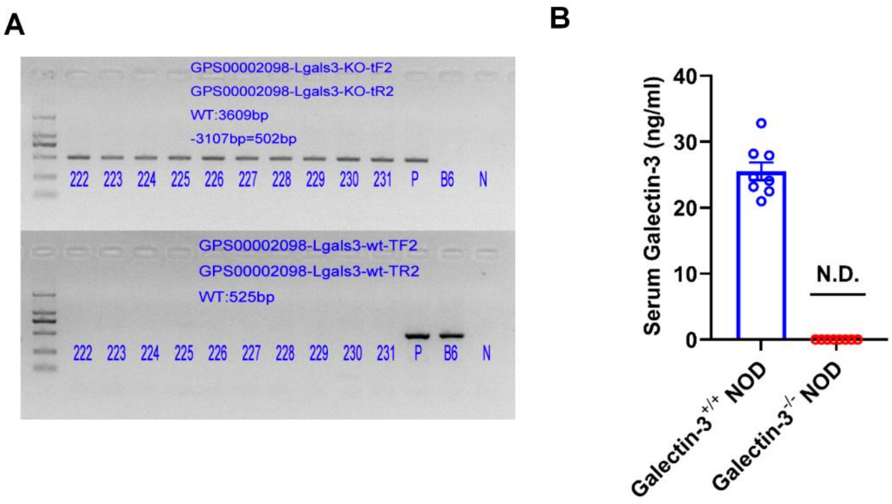


**Fig. S2. Characterization of Galectin-3$^{+/+}$ NOD and Galectin-3$^{-/-}$ NOD mice.** (**A**) Genotyping of Galectin-3$^{-/-}$ NOD mice. (**B**) Serum Galectin-3 levels in Galectin-3$^{+/+}$ NOD and Galectin-3$^{-/-}$ NOD mice. Data are expressed as mean $\pm$ SEM. N. D., not detectable.

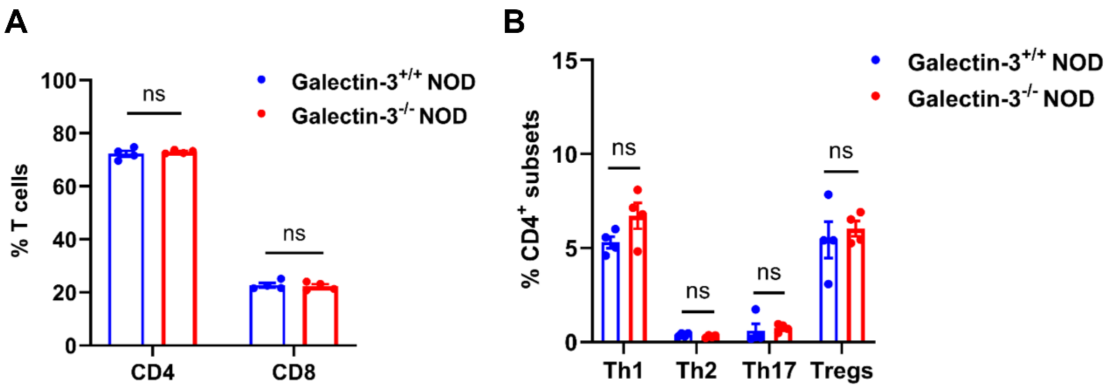


**Fig. S3. Galectin-3 deficiency does not affect T cell subsets in spleen of NOD mice.** Pancreatic islets from 12-week-old non-diabetic Galectin-$3^{+/+}$ NOD and Galectin-$3^{-/-}$ NOD mice were harvested and subject to flow cytometry analysis (n = 4). **(A)** Frequencies of $CD4^{+}$ and $CD8^{+}$ T cells. **(B)** Frequencies of Th1 ($CD4^{+}IFN\text{-}\gamma^{+}$), Th2 ($CD4^{+}IL\text{-}4^{+}$), Th17 ($CD4^{+}IL\text{-}17^{+}$) and Tregs ($CD4^{+}CD25^{+}Foxp3^{+}$) among $CD45^{+}$ cells in islets of 12-week-old Galectin-$3^{+/+}$ NOD and Galectin-$3^{-/-}$ NOD mice. Data are expressed as mean ± SEM. ns, no significance.

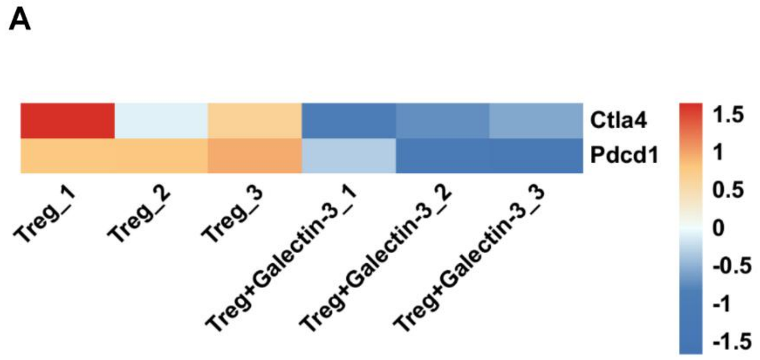


**Fig. S4. Galectin-3 treatment downregulates the mRNA expression of CTLA-4 and PD-1 in Tregs.** (**A**) Heatmap showing the expression levels of *Ctla4* and *Pdcd1* in control Tregs versus Galectin-3–treated Tregs (n = 3).

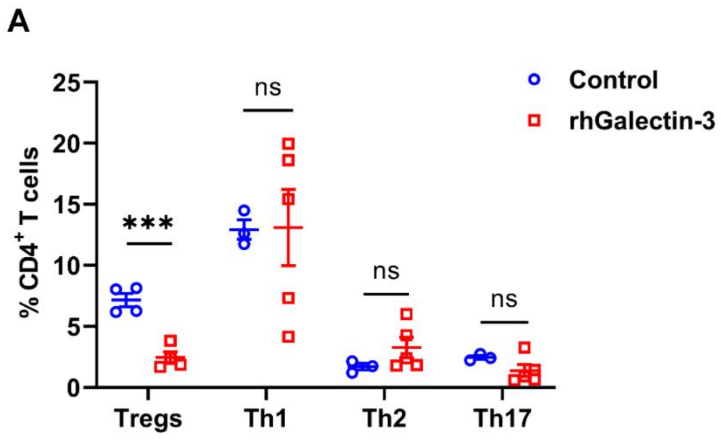


**Fig. S5. Galectin-3 decreases peripheral Treg fraction in human.** (**A**) Frequencies of Tregs ($CD4^+CD25^+Foxp3^+$), Th1 ($CD4^+IFN\text{-}\gamma^+$), Th2 ($CD4^+IL\text{-}4^+$), and Th17 ($CD4^+IL\text{-}17^+$) in total $CD4^+$ T cells from PBMCs of healthy subjects treated with recombinant human Galectin-3 (50 µg/ml) or vehicle for 72 hours (n = 3-5). Data are expressed as mean ± SEM. ns, no significance. *$P < 0.05$.

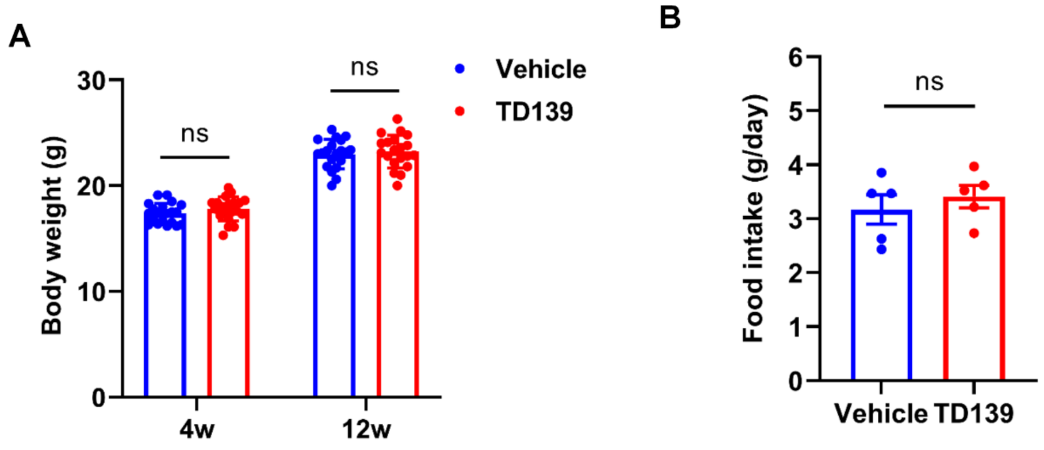


**Fig. S6. Treatment with TD139 does not affect body weight or food intake.** Female 4-week-old NOD mice were treated with TD139 (15 mg/kg·d) or vehicle by intraperitoneal injection for 8 weeks. (**A**) Body weight of NOD mice treated with TD139 or vehicle (n = 20). (**B**) Food intake of NOD mice treated with TD139 or vehicle (n = 5). Data are expressed as mean ± SEM. ns, no significance.

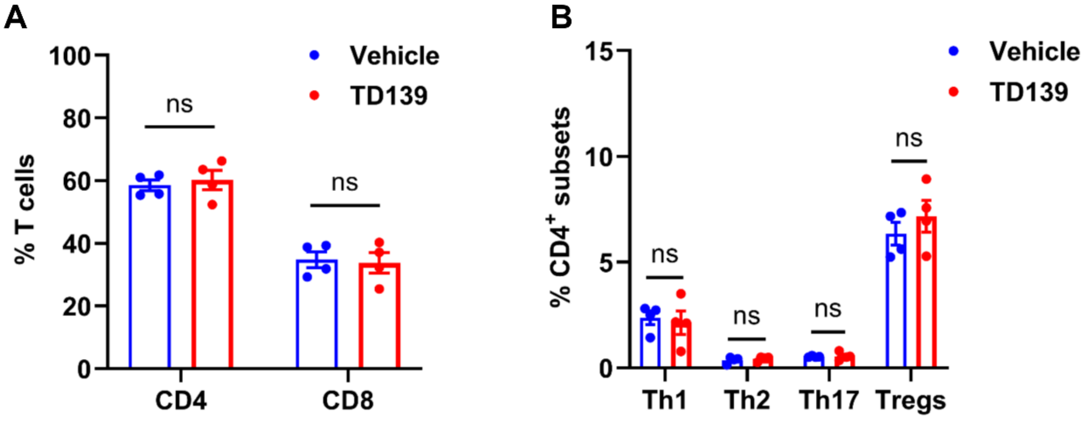


**Fig. S7. Pharmacological inhibition of Galectin-3 does not affect T cell subsets in spleen of NOD mice.** Female 4-week-old NOD mice were treated with TD139 (15 mg/kg·d) or vehicle by intraperitoneal injection for 8 weeks. **(A)** Frequencies of $CD4^+$ and $CD8^+$ T cells. **(B)** Frequencies of Th1 ($CD4^+$IFN-$\gamma^+$), Th2 ($CD4^+$IL-$4^+$), Th17 ($CD4^+$IL-$17^+$) and Tregs ($CD4^+CD25^+Foxp3^+$) among $CD45^+$ cells in islets of 12-week-old NOD mice treated with TD139 or vehicle (n = 4). Data are expressed as mean ± SEM. ns, no significance.

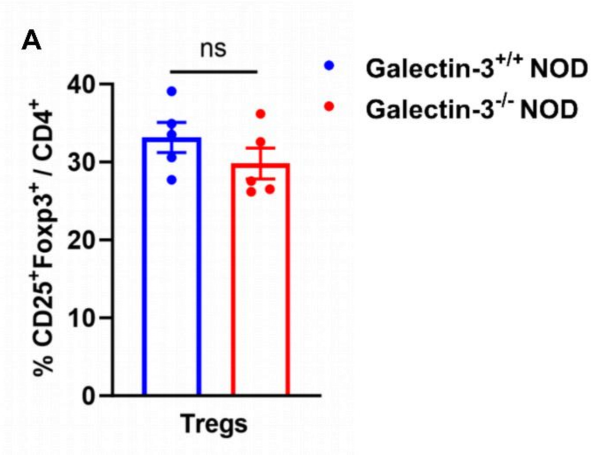


**Fig. S8. Genetic deletion of Galectin-3 in T cells does not affect Treg differentiation in vitro.** **(A)** Frequency of induced Tregs ($CD4^+CD25^+Foxp3^+$) from naïve splenic $CD4^+$ T cells of Galectin-$3^{+/+}$ and Galectin-$3^{-/-}$ NOD mice under Treg-polarizing conditions (n = 5). Data are expressed as mean ± SEM. ns, no significance.

**Table S1. Anthropometric and metabolic data of the study participants.**

| Characteristic | HC (n = 132) | $Ab^-$ FDR (n = 76) | $Ab^+$ FDR (n = 30) | T1D (n = 234) | *P*-value |
|---|---|---|---|---|---|
| Sex (female/male) | 132 (67/65) | 77 (44/32) | 30 (13/17) | 234 (138/96) | 0.238 |
| Age (years) | 15.50 ± 6.53 | 32.56 ± 13.67 [aaa] | 33.15 ± 15.28 [aaa] | 16.57 ± 10.68 [bbbccc] | < 0.001 |
| BMI ($kg/m^2$) | 19.62 ± 3.90 | 21.99 ± 4.05 [aaa] | 21.87 ± 4.94 [a] | 18.58 ± 3.32 [bbbccc] | < 0.001 |
| WHR | 0.83 ± 0.07 | 0.87 ± 0.06 [aa] | 0.90 ± 0.09 [aaa] | 0.83 ± 0.07 [bbccc] | < 0.001 |
| TG (mmol/L) § | 0.84 (0.67, 1.21) | 0.99 (0.64, 1.78) [a] | 0.91 (0.65, 1.32) | 0.70 (0.56, 1.01) [bbb] | < 0.001 |
| TC (mmol/L) | 3.81 ± 0.58 | 4.43 ± 0.80 [aaa] | 4.21 ± 0.71 | 4.25 ± 0.99 [aaa] | < 0.001 |
| LDL-C (mmol/L) | 1.95 ± 0.53 | 1.34 ± 0.38 | 1.89 ± 0.85 | 2.47 ± 2.32 [bbb] | < 0.001 |
| HDL-C (mmol/L) § | 1.41 (1.28, 1.68) | 2.43 (1.94, 2.94) [aaa] | 1.87 (1.25, 2.58) [aabbb] | 1.50 (1.26, 1.72) [bbbccc] | < 0.001 |
| FBG (mmol/L) § | 4.40 (4.12, 4.80) | 4.59 (4.17, 5.09) | 4.45 (4.21, 5.01) | 7.41 (5.25, 10.87) [aaabbbccc] | < 0.001 |
| 2hBG (mmol/L) § | 4.92 (3.85, 5.57) | 5.70 (4.50, 6.99) | 5.75 (5.16, 7.19) | 15.90 (10.50, 21.60) [aaabbbccc] | < 0.001 |
| HbA1c (%) § | 5.20 (5.00, 5.40) | 5.50 (5.30, 5.70) | 5.55 (5.23, 5.80) | 7.50 (6.50, 9.40) [aaabbbccc] | < 0.001 |
| FCP (pmol/L) § | 347.30 (259.30, 409.30) | 420.50 (309.50, 566.30) | 402.90 (227.90, 540.90) | 78.25 (25.25, 165.30) [aaabbbccc] | < 0.001 |
| 2hCP (pmol/L) § | 1098.00 (770.50, 1563.00) | 1819.00 (1287.00, 2402.00) | 1521.00 (905.30, 2026.00) | 167.70 (39.30, 440.20) [aaabbbccc] | < 0.001 |

Abbreviations: HC, healthy controls; $Ab^-$ FDR, islet autoantibodies negative first-degree relatives; $Ab^+$ FDR, islet autoantibodies positive first-degree relatives; T1D, type 1 diabetes; BMI, body mass index; WHR, waist-hip ratio; TG, triglyceride; TC, total cholesterol; LDL-C, low-density lipoprotein-cholesterol; HDL-C, high-density lipoprotein-cholesterol; FBG, fasting blood glucose; 2hBG, 2-hour blood glucose; HbA1c, glycated hemoglobin A1c; FCP, fasting C-peptide; 2hCP, 2-hour C-peptide. Data are mean ± SD, or median (25th - 75th percentile). § Natural logarithm transformed before analysis. [a] *P* compared with HC, [a] $P < 0.05$; [aa] $P < 0.01$; [aaa] $P < 0.001$; [b] *P* compared with $Ab^-$ FDR group, [b] $P < 0.05$; [bb] $P < 0.01$; [bbb] $P < 0.001$; [c] *P* compared with $Ab^+$ FDR group, [c] $P < 0.05$; [cc] $P < 0.01$; [ccc] $P < 0.001$.

**Table S2. Correlation analysis of serum Galectin-3 levels with glucose metabolism indexes and other indexes.**

| Characteristic | HC, FDR and T1D (n = 472) | |
|---|---|---|
| | r | *P* |
| Age | 0.290 | <0.001*** |
| BMI | 0.174 | <0.001*** |
| TG§ | 0.060 | 0.231 |
| TC | 0.178 | <0.001*** |
| LDL-C | 0.044 | 0.378 |
| HDL-C § | 0.093 | 0.062 |
| FBG § | 0.123 | 0.010** |
| 2hBG § | 0.038 | 0.479 |
| HbA1c § | 0.119 | 0.016* |
| FCP § | -0.012 | 0.828 |
| 2hCP § | 0.026 | 0.651 |
| GADA § | -0.015 | 0.812 |
| IA2A § | -0.131 | 0.091 |
| ZnT8A § | -0.134 | 0.112 |

Abbreviations: HC, healthy controls; FDR, first-degree relatives; T1D, type 1 diabetes; BMI, body mass index; TG, triglyceride; TC, total cholesterol; LDL-C, low-density lipoprotein-cholesterol; HDL-C, high-density lipoprotein-cholesterol; FBG, fasting blood glucose; 2hBG, 2-hour blood glucose; HbA1c, glycated hemoglobin A1c; FCP, fasting C-peptide; 2hCP, 2-hour C-peptide; GADA, glutamic acid decarboxylase autoantibody; IA2A, insulinoma-associated protein 2 autoantibody; ZnT8A, zinc transporter 8 autoantibody. § Natural logarithm transformed before analysis. * $P < 0.05$; ** $P < 0.01$; *** $P < 0.001$.

**Table S3. Anthropometric and metabolic data of the study participants for scRNA-seq.**

| Characteristic | HC (n = 5) | T1D (n = 5) | *P*-value |
|---|---|---|---|
| Sex (female/male) | 5 (3/2) | 5 (3/2) | 1.000 |
| Age (years) | 32.60 ± 12.12 | 31.40 ± 10.69 | 0.872 |
| BMI ($kg/m^2$) | 19.85 ± 2.24 | 21.98 ± 2.54 | 0.197 |
| HbA1c (%) | 5.16 ± 0.26 | 7.79 ± 1.14 | $0.001^{**}$ |

Abbreviations: HC, healthy controls; T1D, type 1 diabetes; BMI, body mass index; HbA1c, glycated hemoglobin A1c. Data are mean ± SD. $^{**}$ $P < 0.01$.

**Table S4. List of primers used for genotyping of Galectin-3$^{-/-}$ NOD mice.**

| Primer type | Forward primer (5'-3') | Reverse primer (5'-3') |
|---|---|---|
| Wild type | TGGACCACTGGTAAGAAGGAAGTTG | GGTAAACTGGAAGGCTCCTTGCAT |
| Mutant | TTCTACCACCGAACTTTCCCTTTG | AAGCTAGGATGTTGGGTATCCACC |

**Table S5. List of primers used for real-time PCR.**

| Gene | Forward primer (5'-3') | Reverse primer (5'-3') |
|---|---|---|
| hLGALS3 | GCGGAAAATGGCAGACAATT | CTTGAGGGTTTGGGTTTCCA |
| hACTB | GTGCTATGTTGCTCTAGACTTCG | ATGCCACAGGATTCCATACC |
| m*Lgals3* | CCGCTTCAATGAGAACAACAG | GGTCAGCTTCAACCAGGACTT |
| m*β-actin* | GCAGGAGTACGATGAGTCCG | ACGCAGCTCAGTAACAGTCC |

h stands specific to human; m stands specific to mouse.

**Table S6. Key resources table.**

| Reagent or Antibodies | Source | Identifier |
|---|---|---|
| Rat Anti-Mouse CD16/CD32 | BD Biosciences | Cat# 553141 |
| Fixable Viability Stain 780 | BD Biosciences | Cat# 565388 |
| Alexa Fluor 700 Rat Anti-Mouse CD45 | BD Biosciences | Cat# 560510 |
| BV510 Hamster Anti-Mouse CD3e | BD Biosciences | Cat# 563024 |
| BV786 Rat Anti-Mouse CD4 | BD Biosciences | Cat# 563331 |
| PE-CF594 Rat Anti-Mouse CD4 | BD Biosciences | Cat# 562285 |
| FITC Rat Anti-Mouse CD8a | Biolegend | Cat# 100803 |
| PE-Cy7 Rat Anti-Mouse IFN-γ | BD Biosciences | Cat# 557649 |
| BV650 Rat Anti-Mouse IL-4 | BD Biosciences | Cat# 564004 |
| BV786 Rat Anti-Mouse IL-4 | BD Biosciences | Cat# 564006 |
| PE Rat Anti-Mouse IL-17a | Biolegend | Cat# 506904 |
| APC Rat Anti-Mouse Perforin | Biolegend | Cat# 154404 |
| BV421 Rat Anti-Mouse Granzyme B | Biolegend | Cat# 396414 |
| PE Rat Anti-Mouse CD25 | Biolegend | Cat# 113704 |
| Alexa Fluor 647 Rat Anti-Mouse Foxp3 | BD Biosciences | Cat# 560401 |
| Alexa Fluor 647 Rat Anti-Mouse F4/80 | Biolegend | Cat# 123122 |
| BB515 Rat Anti-Mouse CD11b | BD Biosciences | Cat# 564454 |
| Alexa Fluor 647 Mouse Anti-Insulin | BD Biosciences | Cat# 565689 |
| PE Anti-Mouse/Human Galectin-3 | Biolegend | Cat# 126706 |
| FITC Rat Anti-Mouse Ki-67 Antibody | Biolegend | Cat# 652410 |
| PerCP-Cy5.5 Rat Anti-Mouse LAG3 | Biolegend | Cat# 125211 |
| Human Fc Block | BD Biosciences | Cat# 564220 |
| FITC Mouse Anti-Human CD45 | BD Biosciences | Cat# 561865 |
| PerCP-Cy5.5 Mouse Anti-Human CD3 | BD Biosciences | Cat# 560835 |
| BV421 Mouse Anti-Human CD4 | BD Biosciences | Cat# 562424 |
| PE Mouse Anti-Human CD25 | BD Biosciences | Cat# 555432 |
| BB700 Mouse Anti-Human FoxP3 | BD Biosciences | Cat# 566526 |
| BV711 Mouse Anti-Human IFN-γ | BD Biosciences | Cat# 564793 |
| PE-Cy7 Mouse Anti-Human IL-4 | BD Biosciences | Cat# 560672 |

| | | |
|---|---|---|
| PE Mouse Anti-Human IL-17A | BD Biosciences | Cat# 560487 |
| Compensation Beads | Invitrogen | Cat# 01-2222-42 |
| FITC Annexin V Apoptosis Detection Kit | BD Biosciences | Cat# 556547 |
| CFSE Cell Division Tracker Kit | Biolegend | Cat# 423801 |
| Recombinant Human Galectin-3 Protein | ImmunoDiagnostics | Cat# 41690 |
| Recombinant Mouse Galectin-3 Protein | R&D Systems | Cat# 1197-GA |
| Anti-Mouse IL-10 monoclonal antibody | Biolegend | Cat# 505037 |
| Anti-Mouse TGF-β1 monoclonal antibody | Biolegend | Cat# 521707 |
| Anti-Mouse LAG3 monoclonal antibody | BioXCell | Cat# BE0174 |
| Anti-ERK1 + ERK2 antibody | Abcam | Cat# ab184699 |
| Anti-ERK1 (phospho T202 + Y204) + ERK2 (phospho T185 + Y187) antibody | Abcam | Cat# ab278538 |
| Anti-MEK1 + MEK2 antibody | Abcam | Cat# ab178876 |
| Anti-MEK1 (phospho S221) + MEK2 (phospho S221) antibody | Abcam | Cat# ab278564 |
| β-Tubulin Rabbit monoclonal antibody | Abiowell | Cat# AWA80025 |
| INS Mouse monoclonal antibody | Proteintech | Cat# 66198-1-Ig |
